# Integrated metabolome and transcriptome analysis reveals salicylic acid and flavonoid pathways’ key roles in cabbage’s defense responses to *Xanthomonas campestris* pv. *campestris*

**DOI:** 10.3389/fpls.2022.1005764

**Published:** 2022-10-31

**Authors:** Qingguo Sun, Zhongmin Xu, Wei Huang, Dawei Li, Qi Zeng, Lin Chen, Baohua Li, Enhui Zhang

**Affiliations:** State Key Laboratory of Crop Stress Biology for Arid Area, College of Horticulture, Northwest A&F University, Xianyang, China

**Keywords:** cabbage, black rot, transcriptome, metabolome, SA metabolism, flavonoid

## Abstract

*Xanthomonas campestris* pv. *campestris* (*Xcc*) is a vascular bacteria pathogen causing black rot in cabbage. Here, the resistance mechanisms of cabbage against *Xcc* infection were explored by integrated metabolome and transcriptome analysis. Pathogen perception, hormone metabolisms, sugar metabolisms, and phenylpropanoid metabolisms in cabbage were systemically re-programmed at both transcriptional and metabolic levels after *Xcc* infection. Notably, the salicylic acid (SA) metabolism pathway was highly enriched in resistant lines following *Xcc* infection, indicating that the SA metabolism pathway may positively regulate the resistance of *Xcc*. Moreover, we also validated our hypothesis by showing that the flavonoid pathway metabolites chlorogenic acid and caffeic acid could effectively inhibit the growth of *Xcc*. These findings provide valuable insights and resource datasets for further exploring *Xcc*–cabbage interactions and help uncover molecular breeding targets for black rot-resistant varieties in cabbage.

## Introduction

Cabbage (*Brassica oleracea* var. *capitata* L.) is an economically important vegetable due to its wide adaptability, good storage properties, and high nutritional value (Quiros and Farnham, 2011). Biotic stresses are one of the key factors affecting the production and quality of cabbage, among which black rot is one of the most serious and destructive bacterial diseases present worldwide (Williams, 1980). The symptoms of infected cabbage with black rot are usually accompanied by yellow V-shaped chlorotic lesions along the margins of leaves, together with leaf wilting, vein necrosis, and the occasional death of whole plants (Williams, 1980). Currently, the strategies to control the black rot of cabbage are pesticide spray, crop rotation, and disease-free seeds. The above strategies could reduce black rot infections, but none of them could prevent them. Also, pesticide spray is not an environmentally friendly approach. Molecular breeding is a powerful tool for battling bacterial infections and diseases in crops, especially with the aid of recent rapid progress made by gene editing technologies (Yin and Qiu, 2019). Understanding the molecular mechanisms of these plant–bacterial interactions is the key to molecular breeding efforts to effectively control plant bacterial diseases.

Plant responses to pathogen infections are highly coordinated processes with large-scale reprogramming in gene expression and metabolism (Ranjan et al., 2019; Li et al., 2021). Recent advances in transcriptomics and metabolomics technologies allow for powerful and cost-efficient exploration of global transcriptional and metabolic responses to pathogen infection. The integrated transcriptomics and metabolomics analysis were successfully used to study the plant–pathogen interactions, including *Glycine max* (Ranjan et al., 2019), *Zanthoxylum bungeanum* (Li et al., 2021), *Asparagus officinalis* (Abdelrahman et al., 2020), and *Camellia oleifera* (Yang et al., 2022), and these studies help reveal the molecular mechanisms of plant-pathogen interactions in a system biology approach. Although recent studies have advanced our understanding of *Xcc*–plant interactions (Wang et al., 2015; Santos et al., 2019; Song et al., 2022), the molecular mechanisms of plant resistance against this pathogen are still limited. We have previously studied the transcriptional regulation of cabbage in response to *Xcc* during the early infection stage by transcriptomic analysis, but the lack of the control plants in the study limits our ability to reveal truly significant expressed genes as well as the molecular pathways in cabbage’s responses to *Xcc* infection (Sun et al., 2021).

Therefore, to gain a deeper understanding of *Xcc*–cabbage interaction mechanisms, we used integrated metabolomic and transcriptomic approaches with well-designed control plants in a time-course experimental design to study cabbage’s defensive responses to *Xcc* infection. There were a large number of differentially expressed pathogen-response genes and differentially accumulated metabolites were identified between resistant and susceptible lines, allowing us to develop a working model of cabbage’s response to *Xcc* infection. Importantly, we could test our hypothesis by wet-lab experiments that two flavonoid pathway metabolites, chlorogenic acid and caffeic acid, could effectively inhibit the growth of *Xcc*. To our knowledge, this work is the first to integrate widely targeted metabolomic and transcriptomic technologies to study how cabbage responds to black rot infection, and the metabolic profiling data, together with the integrated transcriptomic data and the validated anti-bacterial metabolites, not only help uncover molecular mechanisms of cabbage–*Xcc* interactions but also serve as valuable resources for the research community to further explore.

## Materials and methods

### Plant material and pathogen inoculation

Two DH lines of cabbage, one resistant QP07 (R) and one susceptible DBP71 (S), were used in this study (Sun et al., 2021). These two lines were obtained by microspore culture using a moderately resistant variety to black rot followed by multiple generations of self selection. Plants were grown in a growth chamber under 16 h light and 8 h dark photoperiod cycles at the Northwest A&F University, Xianyang, China. *Xcc* strains were grown on a nutrient agar (NA) medium at 28°C. The bacterial suspension was prepared to a density of 1 × 10^8^ cfu/ml in distilled water after cultivation. Forty-five-day-old cabbage seedlings were infected with *Xcc* by spraying the leaves with the bacterial suspension. Control plants were inoculated with distilled water. During the *Xcc* infection assay, the inoculated seedlings were maintained in a growth chamber under a 16-hour photoperiod at 28°C and kept moist during the treatment. Plant leaves were sampled at three-time points (2, 7, and 12 days post-inoculation, dpi). For each time point, three to five plants per replicate were sampled (three biological replicates). Following collection, samples were immediately frozen in liquid nitrogen and stored at −80°C prior to RNA extraction and metabolomic analysis.

### Metabolites estimation and analysis

The extraction method was following the MetWare Biological Science and Technology Co., Ltd. (Wuhan, China) standard protocol. Metabolite profiling was carried out using the UPLC-ESI-MS/MS system (UPLC, Shim-pack UFLC Shimadzu CBM30A system; MS, Applied Biosystems 4500 Q TRAP) by comparing the fragmentation patterns, the retention time, and the accurate m/z value to the standards in the MetWare self-compiled database and the public databases.

Metabolite data for samples with and without *Xcc* infection analysis were conducted with the Analyst 1.6.1 software (AB SCIEX, Ontario, Canada). The Pearson correlation coefficients analysis, principal component analysis (PCA) and orthogonal partial least squares discrimination analysis (OPLS-DA) of identified metabolites between samples were carried out by the corresponding functions in the R package. We used the variable importance in projection (VIP) score obtained by the OPLS-DA model to obtain the maximum differences between control and *Xcc* infection. Metabolites with VIP ≥1.0 and fold changes ≥1.5 or ≤0.67 were determined as differentially accumulated metabolites (DAMs). The KEGG database was used to annotate and display the differential metabolites that are correlated with the resistance of cabbage to *Xcc* infection.

### Total RNA extraction and RNA-seq analysis

Total RNA was extracted using the TRIzol reagent (Invitrogen, Carlsbad, CA, USA) according to the manufacturer’s protocol. The concentration, quality, and integrity of the extracted RNA were evaluated with 1% agarose gel electrophoresis and an Agilent Bioanalyzer 5400 (Agilent Technologies, Santa Clara, CA, USA). The RNA samples that met the requirements were sent to MetWare for cDNA library construction and transcriptome sequencing on an Illumina HiSeq 2000 system. Clean data were obtained by filtering and quality-controlling the raw data using the FastQC tool (http://www.bioinformatics.babraham.ac.uk). The clean data were uniquely mapped to the cabbage genome (*B. oleracea* cv. JZS V2.0, http://brassicadb.cn). The gene expression levels were estimated as fragments per kilobase per transcript per million mapped reads (FPKM). Differentially expressed genes (DEGs) were identified from the comparison of inoculated cabbages of both lines at different time points to their respective controls by using the DEseq R package if the FDR was <0.05 and |log2(fold change)| was ≥1. The DEGs were further aligned with six databases: GO (Gene Ontology), KEGG (Kyoto Encyclopedia of Genes and Genomes), NR (NCBI non-redundant protein sequences), KOG (a cluster of orthologous groups), Pfam (protein family), and Swiss-Prot (a manually annotated and reviewed protein sequence database).

### Anti-bacterial activities of chlorogenic acid and caffeic acid on *X. campestris*

To examine the effects of chlorogenic acid (95%, Aladding) and caffeic acid (99%, Aladding) on the growth of *Xcc*, the *Xcc* bacterial suspension with a density of 1 × 10^8^ cfu/ml was diluted 2,000 times with sterilized water, and 50 μl of the dilution suspension were spread on the 90 mm petri dish containing 15 ml of NA medium (with agar). Next, we added the bacterial suspension to the NA medium (without agar) at a ratio of 1:2,000. The final applied concentrations of these metabolites in the NA medium were 0.5, 0.25, and 0.1 mM, respectively. Each treatment was composed of three replicates. The bacterial concentrations were calculated after 24 and 48 h of incubation at 28°C.

### Validation of gene expression from transcriptome data

The RNA-sequencing results were confirmed by qRT-PCR of 15 candidate DEGs. The sampled leaves of cabbage were the same as those for transcriptome analysis. Three biological replicates were used for each treatment. The DEG-specific primers for qRT-PCR were designed by Primer3 web (http://primer3.ut.ee/) for the amplification of gene fragments of approximately 100–200 bp in length (**Supplementary Table 1**). qRT-PCR was performed on an ABI QuantStudio 5 real-time PCR detection system (ThermoFisher Scientific, USA) using a Hieff^®^ qPCR SYBR Green Master Mix. The total volume for qRT-PCR was 25 μl and procedures were as follows: one cycle at 95°C for 5 min, 40 cycles at 95°C for 10 s, and 60°C for 30 s. The relative quantitative method of 2^−ΔΔCt^ was employed to calculate the target gene expression levels, which were then analyzed in Excel.

## Results

### Phenotypes of the resistant and susceptible lines after *X. campestris* infection

**Figure 1:**
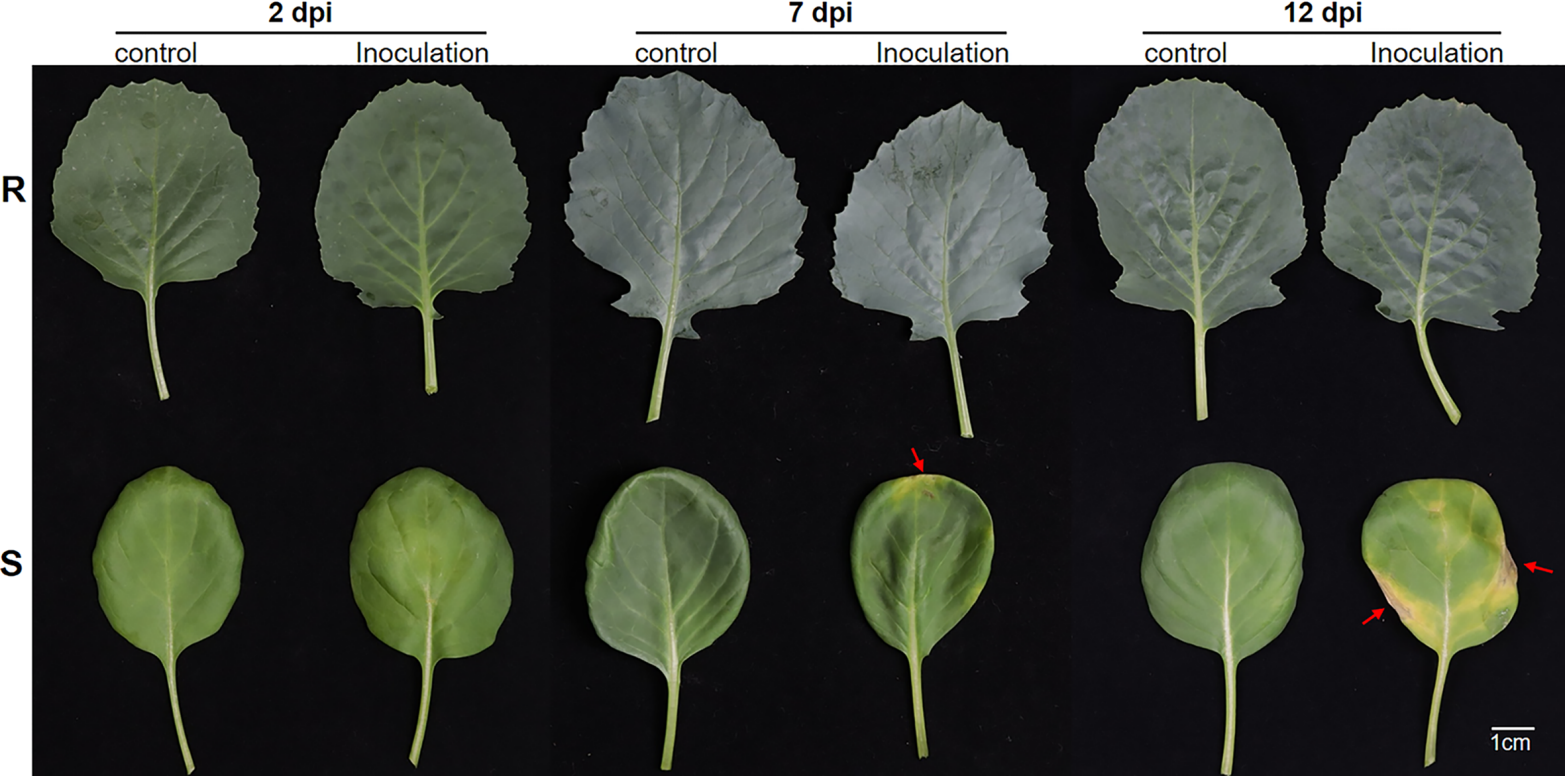
The phenotypes of the R and S cabbage lines in *X. campestris* infection assay. Representative leaves of the R and S cabbage lines infected by *X. campestris* at 2, 7, and 14 days were shown. R, *Xcc*-resistant cabbage line; S, *Xcc*-susceptible cabbage line; dpi, Days post inoculation; Red arrows indicate the symptoms of black rot.

The phenotypic differences during the infection of *Xcc* between cabbage R and S lines in this study were shown (**Figure 1**). There were no disease symptoms in the leaves at 2 dpi in either line. At 7 dpi, typical V-shaped chlorosis occurred around the leaf edge in the S line, while no disease symptoms were detected in the R line or control line. At 12 dpi, the S line had severe V-shaped chlorosis around the leaf, whereas the R line still had no visible black rot symptoms.

### Analysis of metabolite profiling in cabbage lines responsive to *X. campestris* infection

**Figure 2:**
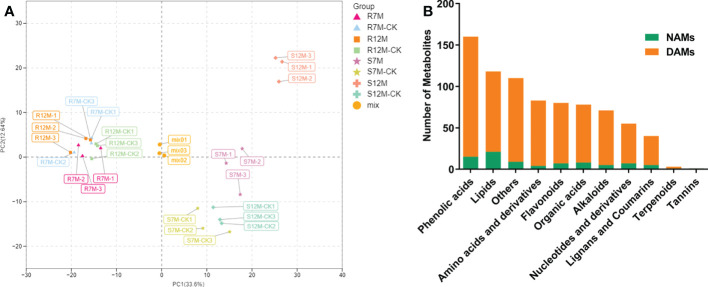
Overview of metabolome analysis of R and S lines in response to *X. campestris* infection. **(A)** PCA of the variance-stabilized estimated raw counts of differentially accumulated metabolites. **(B)** Category and number of metabolites identified in the tested samples. DAMs, Differentially accumulated metabolites; NAMs, Non-differentially accumulated metabolites.

The widely targeted metabolomics pipeline was used for the metabolic profiling of the *Xcc*-infected plant leaf samples from our resistant and susceptible cabbage lines on 7 and 12 dpi, labeled as R7M-CK/R7M, R12M-CK/R12M, S7M-CK/S7M, and S12M-CK/S12M. A total of 799 metabolites were identified in the tested samples, and correlation analysis of the detected metabolites indicated high consistency of the samples, confirming the reliability of the dataset (**Supplementary Figure 1**). The PCA of detected metabolites showed distinct metabolomic profiles at each time point during the course of infection between the R and S lines (**Figure 2A**). The four types of samples from the S line, S7M-CK, S7M, S12M-CK, and S12M, were clearly separated between inoculation and controls, and each separate group was strictly composed of the three biological repeats of that group, confirming the high-quality of the sample identities and metabolic data. However, much smaller internal variations are present among all the samples from the R line compared with the variations from samples of the S line. This supports the observation that the R lines show much smaller phenotypic perturbation after *Xcc* infection compared with the S line.

**Figure 3:**
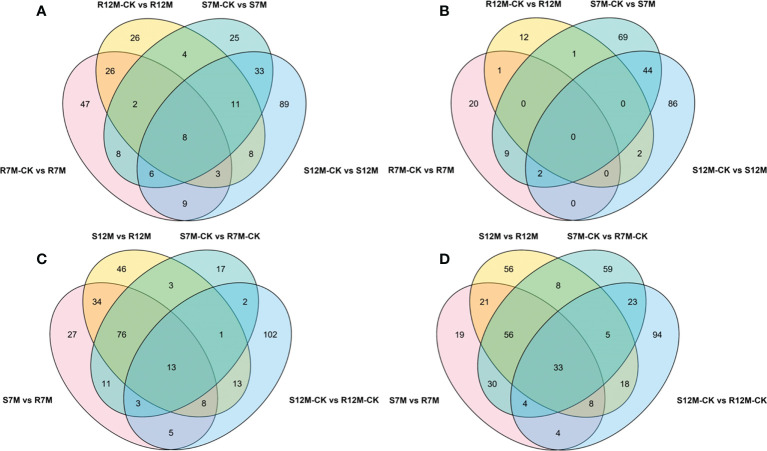
Venn graph for up and downregulated DAMs from the pairwise comparisons. **(A)** Upregulated DAMs in *Xcc*-infected samples of the two cabbage lines compared with the control samples at 7 and 12 dpi; **(B)** Downregulated DAMs in *Xcc*-infected samples of the two cabbage lines compared with the control samples at 7 and 12 dpi; **(C)** Upregulated DAMs in the R line compared with the S line at 7 and 12 dpi; **(D)** Downregulated DAMs in the R line compared with the S line at 7 and 12 dpi.

**Figure 4:**
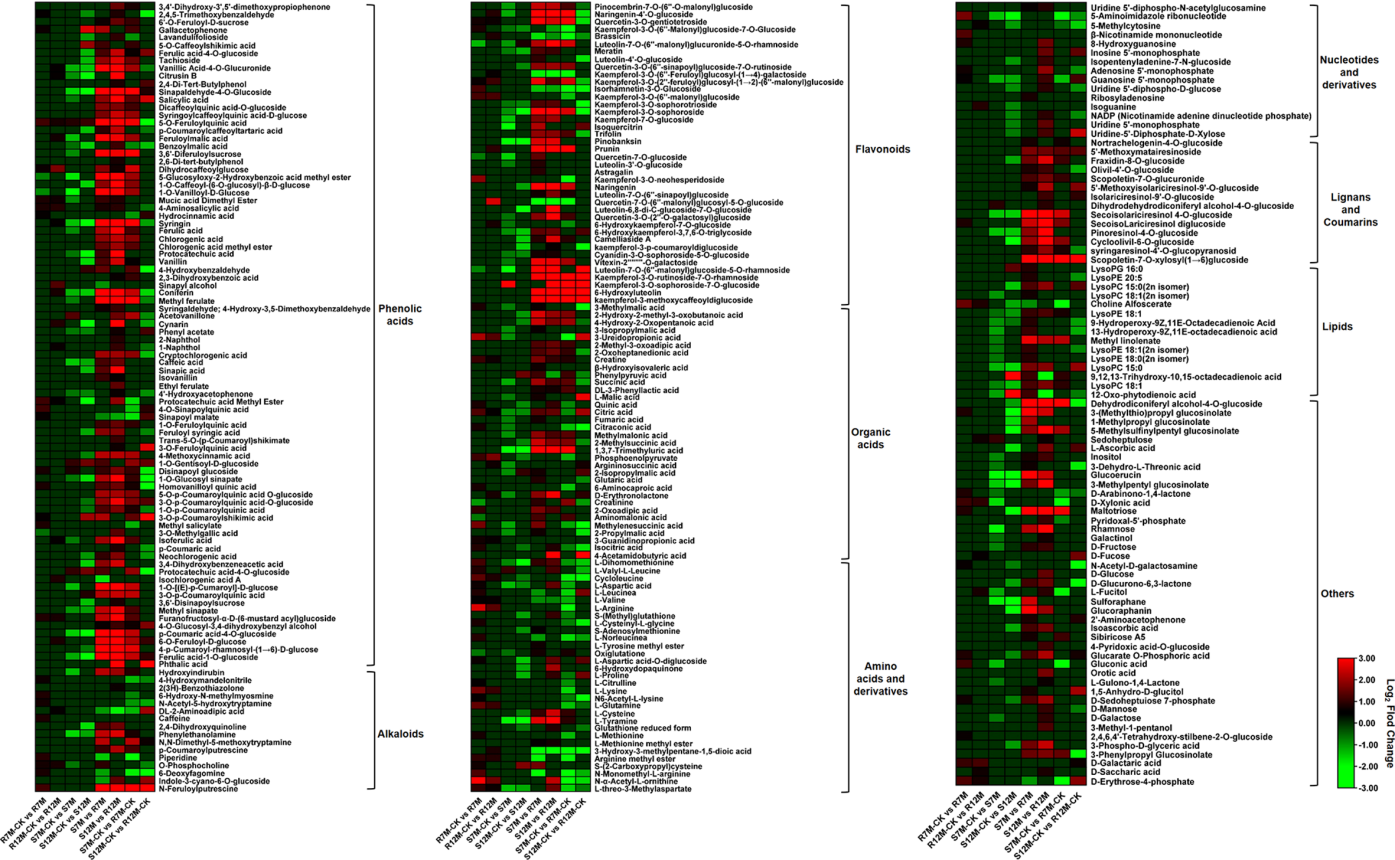
Heatmaps of differentially accumulated metabolites for putative candidate metabolites responsible for the resistance of the R line against *X. campestris* infection. Red bars indicate upregulated DAMs; Green bars indicate downregulated DAMs; The closing parentheses indicate the categories of metabolites.

**Figure 5:**
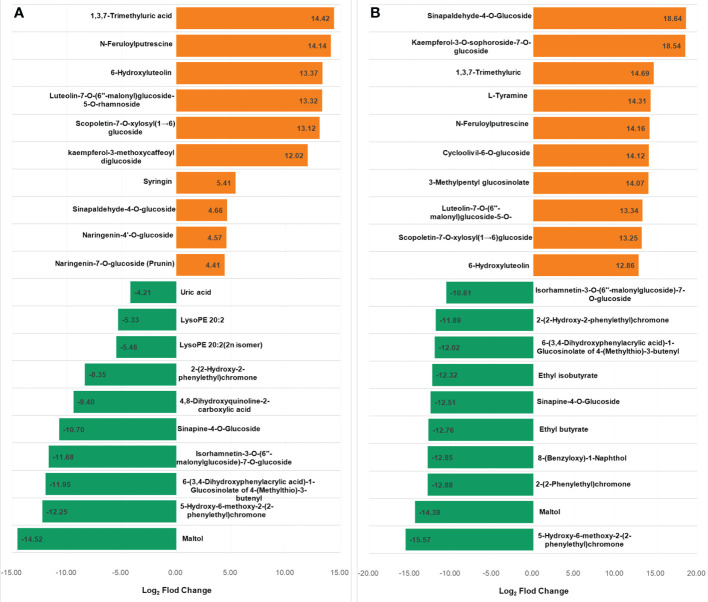
Top 10 up and downregulated DAMs in *X. campestris* infected samples of the R line compared with the *X. campestris* infected samples of the S line. **(A)** Seven days after *Xcc* infection; **(B)** Twelve days after *Xcc* infection; Orange bars indicate upregulated DAMs, and green bars indicate downregulated DAMs.

The metabolites in the dataset could be categorized into 11 classes, including 160 phenolic acids, 118 lipids, 83 amino acids and derivatives, 80 flavonoids, 78 organic acids, 71 alkaloids, 55 nucleotides and derivatives, 40 lignans and coumarins, 3 terpenoids, 1 tannin, and 110 others (**Figure 2B**). After the differentially accumulated analyses, a total of 716 DAMs were detected (**Supplementary Table 2**). Among them, 205 DAMs were identified in *Xcc*-infected samples of the R line compared with the control samples of the R line, 413 DAMs were identified in *Xcc*-infected samples of the S line compared with the control samples of the S line, and 673 DAMs were identified in R line compared with S line. A heatmap of DAMs confirmed that significant biochemical changes occurred after *Xcc* infection (**Supplementary Figure 2**). As shown in the Venn graph, 99 upregulated and 33 downregulated DAMs were uniquely identified in the R line, while 147 upregulated and 199 downregulated DAMs were uniquely identified in the S line (**Figures 3A, B**). Based on comparisons between R and S lines, a total of 240 upregulated DAMs were found in two *Xcc*-inoculated comparisons, while 262 DAMs were downregulated (**Figures 3C, D**). These DAMs might be contributing to the resistance of the R line against *Xcc* infection. The heatmap showed 99 and 240 upregulated metabolites (**Figure 4**), among which the phenolic acids and flavonoids were the most abundant. Furthermore, we identified the top ten upregulated and downregulated DAMs in *Xcc*-infected samples of the R line compared with the *Xcc*-infected samples of the S line (**Figure 5**). Five flavonoid metabolites: 6-hydroxyluteolin, luteolin-7-O-(6’’-malonyl)-glucoside-5-O-rhamnoside, kaempferol-3-methoxycaffeoyldiglucoside, naringenin-4’-O-glucoside, and naringenin-7-O-glucoside were classified into the top 10 upregulated DAMs at 7 dpi. After 12 days of inoculation, three flavonoid metabolites were identified: Kaempferol-3-O-sophoroside-7-O-glucoside, luteolin-7-O-(6’’-malonyl)-glucoside-5-O-rhamnoside and 6-Hydroxyluteolin were identified, supporting that flavonoids might be contributing to the immune responses of the R line against *Xcc* infection.

KEGG pathway analysis of DAMs between the R and S lines showed that six common KEGG pathways were enriched at 7 dpi and 12 dpi (**Supplementary Figure 3**), including “Flavonoid biosynthesis” and “Phenylpropanoid biosynthesis” pathways. We also found the “Flavone and flavonol biosynthesis” pathway enriched at 7 dpi and the “Plant hormone signal transduction” pathway enhanced at 12 dpi.

### RNA-seq analysis and identification of differentially expressed genes in cabbage during *X. campestris* infection

In order to evaluate the molecular mechanisms associated with cabbage resistance to black rot, global transcriptome changes of leaf samples on Days 2, 7, and 12 after infections were used for RNA-seq analysis, labeled as R2T-CK/R2T, R7T-CK/R7T, R12T-CK/R12T, S2T-CK/S2T, S7T-CK/S7T, and S12T-CK/S12T. Importantly, all the leaf samples for RNA-seq analysis were from the same leaf sample pools as the widely targeted metabolic study, ensuring the high quality of the dataset for integrated metabolic and transcriptomic studies. Approximately 43.4–76.6 million raw reads were generated from the 36 tested samples. After quality control, 253.21 Gb of clean data were included. The GC content percentage was approximately 46.71%–48.87% of these reads, and the Q30 quality scores of reads were above 93.97%, suggesting that the accuracy and reliability of sequencing data were reliable enough for further studies (**Supplementary Table 3**). The percentage of reads mapped to the reference cabbage genome was almost the same for R (93%) and S (93.09%). On average, 88.73% of these mapped reads were uniquely mapped to the genome, and 56.41%–68.47% of these reads were mapped to exons (**Supplementary Table 3**).

**Figure 6:**
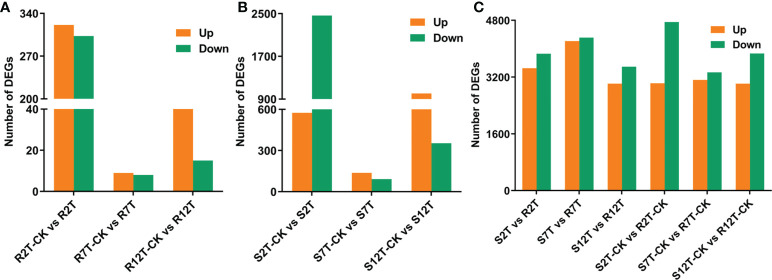
Bar graph of up and downregulated genes from pairwise comparisons. **(A)** Number of DEGs in *Xcc*-infected samples of the R line compared with the control samples of the R line at 2, 7, and 12 dpi; **(B)** Number of DEGs in *Xcc*-infected samples of the S line compared with the control samples of the S line at 2, 7, and 12 dpi; **(C)** Number of DEGs in the R line compared with the S line at 2, 7, and 12 dpi.

**Figure 7:**
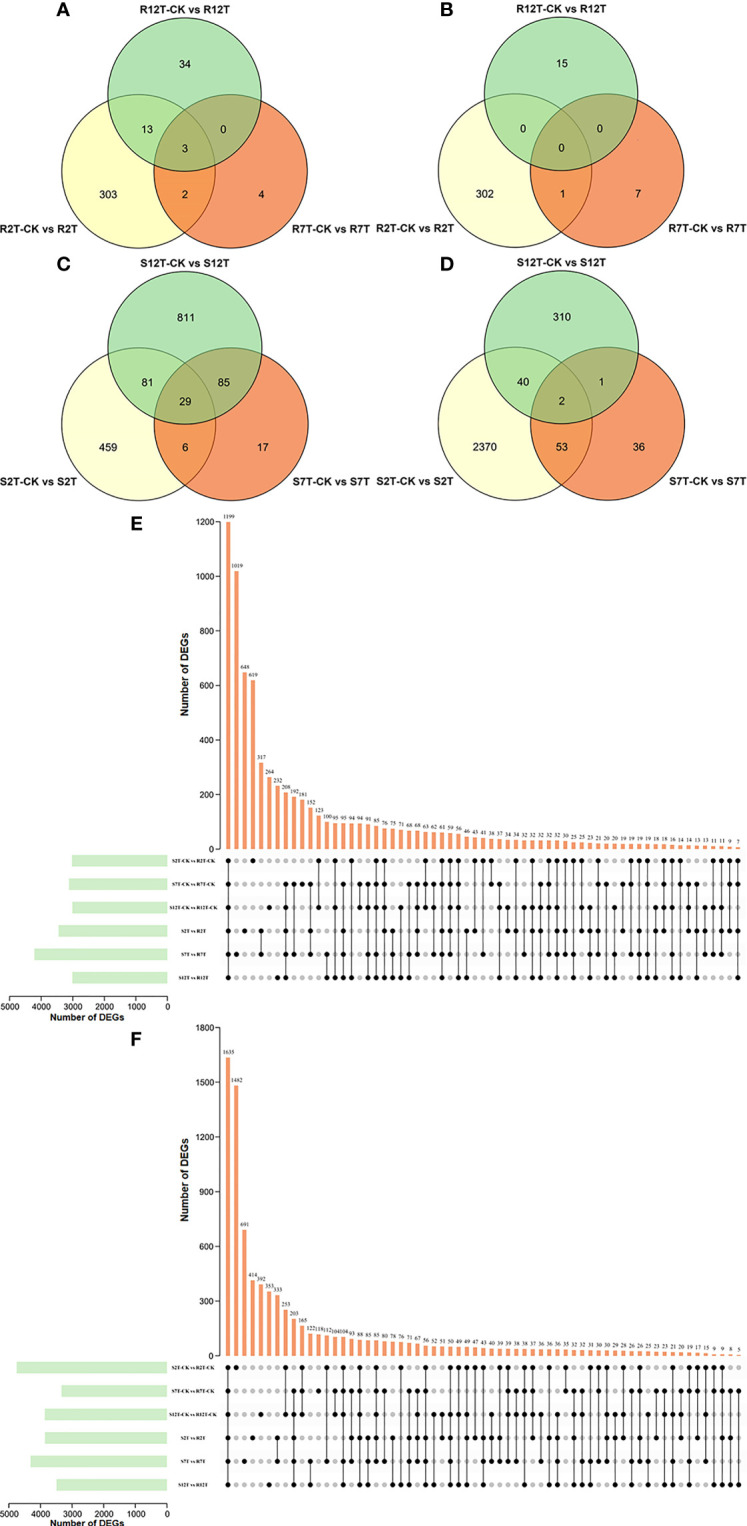
Venn graph for up and downregulated DEGs from the pairwise comparisons. **(A)** Upregulated DEGs in *Xcc*-infected samples of the R line compared with the control samples of the R line at 2, 7, and 12 dpi; **(B)** Downregulated DEGs in *Xcc*-infected samples of the R line compared with the control samples of the R line at 2, 7, and 12 dpi; **(C)** Upregulated DEGs in *Xcc*-infected samples of the S line compared with the control samples of the S line at 2, 7, and 12 dpi; **(D)** Downregulated DEGs in *Xcc*-infected samples of the S line compared with the control samples of the S line at 2, 7, and 12 dpi; **(E)** Upregulated DEGs in the R line compared with the S line at 2, 7, and 12 dpi; **(F)** Downregulated DEGs in the R line compared with the S line at 2, 7, and 12 dpi. Black circles indicate common DEGs for different comparisons.

A total of 15,980 DEGs in the 12 comparison groups were identified by using DESeq with FPKM values. In infected samples of the R line, 321 genes were upregulated and 303 genes were downregulated at 2 dpi compared with the control samples of the R line, while only nine genes were upregulated and eight genes were downregulated at 7 dpi, and 50 genes were upregulated and 15 genes were downregulated at 12 dpi (**Figure 6A**). The total number of upregulated and downregulated genes in the R line is similar, being over-represented at 2 dpi. In the S line, the three comparisons (S2T-CK *vs* S2T, S7T-CK *vs* S7T, and S12T-CK *vs* S12T) generated 575, 137, and 1,006 upregulated genes, as well as 2,465, 92, and 353 downregulated genes, respectively (**Figure 6B**). Most of the genes of *Xcc*-infected samples in the S line were downregulated at 2 dpi and upregulated at 12 dpi compared with the control samples of the S line, indicating that the defense response genes in the S line after *Xcc* infection were not induced successfully during the early infection stage, which might result in the *Xcc* susceptible phenotypes of the S line. Moreover, the number of DEGs in the infected S line was significantly higher than the one in the infected R line, and these transcriptomic data are consistent with the metabolomic finding that the metabolites after *Xcc* infection in the R line are more stable than the ones in the S line (**Figure 2**). To explore the molecular mechanisms of how the R and S lines respond to *Xcc* infection (**Figure 6C**), we found that 3,451 genes were upregulated and 3,858 genes were downregulated in *Xcc*-infected samples of the R line compared with the *Xcc*-infected samples of the S line at 2 dpi; 4,215 genes were upregulated and 4,313 genes were downregulated at 7 dpi; 3,013 genes were upregulated and 3,493 genes were downregulated at 12 dpi. The above results indicated that there were significant changes in gene expression levels between the R and S lines following *Xcc* infection. Then we performed Venn diagram analyses for different comparisons (**Figure 7**). Only three common genes were upregulated and no genes were downregulated at all tested time points in *Xcc*-infected samples of the R line compared with the control samples of the R line. Twenty-nine genes were upregulated and two genes were downregulated in *Xcc*-infected samples of the S line compared with the control samples of the S line. We also analyzed DEGs between the R and S lines. A total of 1,199 genes were upregulated and 1,635 genes were downregulated in six comparisons at all three-time points, while 75 genes were upregulated and 85 genes were downregulated in only three *Xcc*-inoculated comparisons.

### Functional annotation of DEGs in cabbage lines responsive to *X. campestris* infection

The 15,980 DEGs between the 12 comparison groups were aligned by comparing their sequences against GO, KEGG, NR, KOG, Pfam, and Swiss-Prot public databases (**Supplementary Table 4**). Based on the GO terms, 684 DEGs in the three comparisons of the R line were assigned to three GO classes: biological process, cellular component, and molecular function, with 22, 16, and 10 categories, respectively (**Supplementary Figure 4A**). In the S line, 4,271 DEGs were assigned to 26 biological process categories, 18 cellular component categories, and 12 molecular function categories, respectively (**Supplementary Figure 4B**). We also analyzed the GO annotation of DEGs at three-time points between the R and S lines after inoculation. A total of 11,737 DEGs were assigned to 28 categories of biological process, 16 categories of cellular components, and 12 categories of molecular function (**Supplementary Figure 4C**). GO enrichment analysis of the DEGs in the biological process categories was analyzed to evaluate the response of cabbage to *Xcc* infection (**Supplementary Table 5**). Our data showed that the “response to reactive oxygen species” and “response to toxic substance” categories were present in all nine comparisons of R-CK *vs* R; S-CK *vs* S; and S *vs* R. We also found that “phenylpropanoid-related process,” “flavonoid metabolic-related process,” “plant hormone metabolic related process,” “defense response to Gram-negative bacterium,” and “immune response related process” to these comparisons, supporting our findings above made by metabolomic analysis.

To explore the enriched biological pathways, we extracted the top 50 enriched KEGG pathways across all nine comparisons (**Supplementary Figure 5**). Among these, we found that “MAPK signaling pathway–plant” and “Phenylpropanoid biosynthesis” were present in these comparisons. We focused on direct comparisons between the R and S lines, and there are 28 KEGG pathways commonly present in all three comparisons, including “plant–pathogen interaction” and “plant hormone signal transduction.” Moreover, the “flavonoid biosynthesis” pathway was enriched in both 2 dpi and 7 dpi between the R and S lines, highlighting the key role that the flavonoid biosynthesis pathway plays in cabbage’s defense responses to *Xcc* infection.

### DEGs and DAMs involved in resistance to *X. campestris*

**Figure 8:**
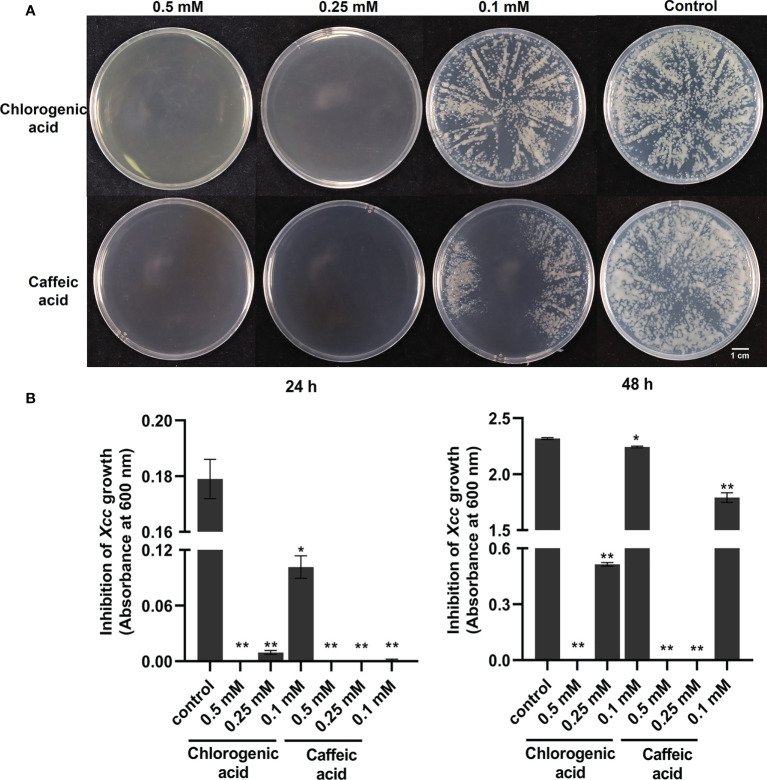
Anti-bacterial activities of flavonoid pathway metabolisms chlorogenic acid and caffeic acid on the growth of *X*. *campestris*. **(A)** Effects of chlorogenic acid and caffeic acid on the growth of *Xcc* grown on plates for 48 h; **(B)** Effects of chlorogenic acid and caffeic acid on the concentration of *Xcc* in liquid medium at 24 h and 48 h. *: p < 0.05, **: p < 0.01 by t-test.

With the above integrated metabolic and transcriptomic analysis, we tried to further combine the large data and analysis together. We also proposed a working hypothesis of how cabbage responds to *Xcc* infection. A list of differentially regulated disease-resistance genes and metabolites was obtained from our data (**Supplementary Tables 2**, **6**). We focused on the profile of DEGs and DAMs associated with plant hormones in *Xcc*-infected samples of the R line compared with the *Xcc*-infected samples of the S line, especially SA, JA, and ET. Twenty-one genes (including 19 upregulated and two downregulated) were related to the SA metabolism pathway. Thirty-four JA metabolism-related genes were downregulated. Twelve genes (including 11 upregulated and one downregulated) were related to the ET metabolism pathway. These DEGs include the synthesis, degradation, and transcriptional regulation of corresponding plant hormone pathways. In addition, we found the accumulation of SA was upregulated and its degraded metabolite SAG was downregulated in *Xcc*-infected samples of the R line compared with the *Xcc*-infected samples of the S line. We also found that 16 genes (including 11 upregulated and five downregulated) were related to the phenylpropanoid and flavonoid metabolism pathway. Furthermore, most of the phenylpropanoid and flavonoid pathway-related metabolites were upregulated in *Xcc*-infected samples of the R line compared with the *Xcc*-infected samples of the S line. We also investigated and validated the inhibitory effects of two flavonoid pathway metabolites, chlorogenic acid and caffeic acid, on *Xcc* growth. The growth of *Xcc* was significantly inhibited by three different concentrations of chlorogenic acid and caffeic acid, respectively (**Figure 8**). Among these, 0.5 mM chlorogenic acid and 0.5 mM and 0.25 mM caffeic acid can completely inhibit the growth of *Xcc* at 24 h and 48 h.

**Figure 9:**
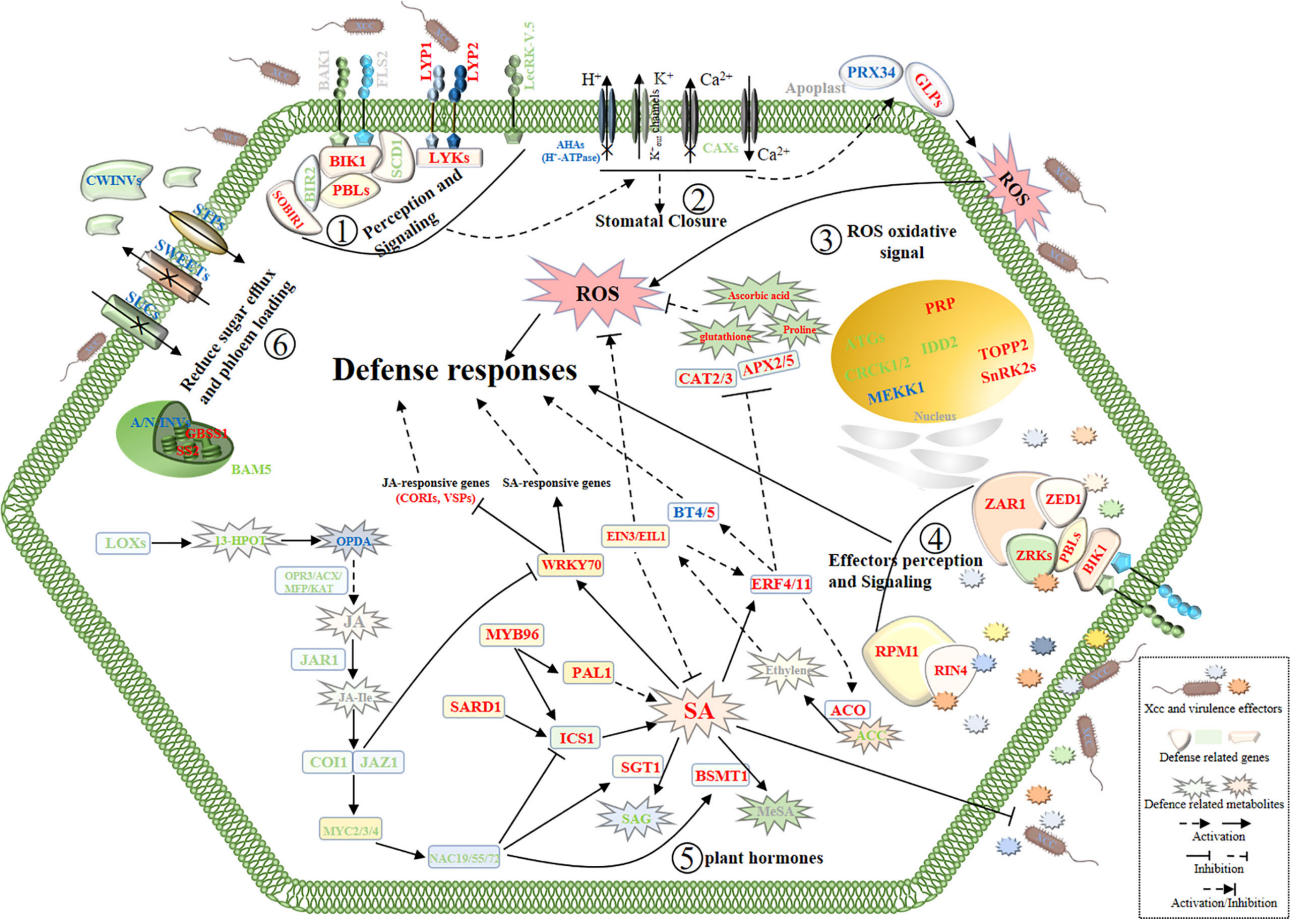
Schematic view of a working model of cabbage’ defense responses to *X*. *campestris* infection with the highlighted DEGs and DAMs from the integrated metabolome and transcriptome analysis. The names in red and green indicate genes or metabolites with up and downregulated, respectively. Blue symbols indicate homologous genes with both up and downregulated. Gray symbols indicate genes and metabolites that were not annotated. SA, Salicylic acid; JA, Jasmonic acid; JA-Ile, Jasmonic acid-isoleucine. The plant defense responses begin with the recognition of the pathogen at the surface of cell by receptor proteins; (Step 1), then stomata closure and oxidative stress responses occur (Steps 2 and 3);finally, activation of defense responses induced by *Xcc* virulence effectors (Step 4) and reprogramming of hormone metabolism and sugar pathways (Steps 5 and 6) work together to battle the invading pathogens.

Moreover, in the pathogen perception and signaling pathway, we found most of the signaling receptor or co-receptor genes were upregulated and several defense-negative regulator genes were downregulated in *Xcc*-infected samples of the R line compared with the *Xcc*-infected samples of the S line. In the stomatal defense pathway, most genes were downregulated in *Xcc*-infected samples of the R line compared with the *Xcc*-infected samples of the S line, including *LecRK-V.5*, *AHA3*, *10*, *11*, and *CAX1*, *2*, *3*, *5* genes. In the pathogen defense-related ROS regulation pathway, most genes were upregulated in *Xcc*-infected samples of the R line compared with the *Xcc*-infected samples of the S line, including *PRX34*, *GLP1*, *HUP39*, *CAT2*, *CAT3*, *APX2*, and *APX5* genes. Moreover, proline, ascorbic acid, and glutathione antioxidant metabolites were upregulated in *Xcc*-infected samples of the R line compared with the *Xcc*-infected samples of the S line. For the defense response of cabbage induced by *Xcc* effectors, we found *ZAR1*, *ZED1*, *ZRK1*, *ZRK4*, *RPM1*, and *RIN4* were upregulated in *Xcc*-infected samples of the R line compared with the *Xcc*-infected samples of the S line. We also found that the expression of some sugar-related genes changed during *Xcc* infection. Thirty DEGs (including 11 upregulated and 19 downregulated) were related to the sugar metabolism pathway. To get a global view of DEGs involved in resistance to *Xcc*, we propose a working model of how these genes and metabolites are responding and coordinating together in cabbage’s responses to *Xcc* infection (**Figure 9**).

### qRT-PCR verification of gene expression

To confirm the transcriptomic results, qRT-PCR was conducted on representative DEGs from ROS oxidative signal, SA metabolism pathways, JA signaling pathways, ET signaling pathways, and flavonoid metabolism pathways. For all 15 genes, the expression profiles shown by the qRT-PCR were similar to the fold change data in the RNA-Seq analysis (**Supplementary Figure 6**), confirming the reliability of our RNA-Seq dataset.

## Discussion

### Perception and signaling of cabbage to pathogen during *X. campestris* infection

Plant defense responses generally start with the detection of pathogen-associated molecular patterns (PAMPs) by pattern recognition receptor (PRR) proteins located on the external surface (Jones and Dangl, 2006). Similarly, we found that some of the *PRRs* genes were significantly induced in *Xcc*-infected samples of the R line but not in *Xcc*-infected samples of the S line (**Figure 9**, **Supplementary Table 6**).

The above-detected DEG of *PRRs* has been previously shown to be involved in plants’ innate immunity responses. Functional analysis indicated that BIR2 affects the formation of complexes BAK1-FLS2 by interacting with BAK1, thereby negatively regulating plant innate immunity (Halter et al., 2014). SOBIR1 is involved in BAK1-mediated immune signaling as a suppressor of BIR1, which constitutively activates immune responses when overexpressed in plants (Gao et al., 2009; van der Burgh et al., 2019). A recent study found that BIR2 and SOBIR1 could interact with LPS from *Xcc* and participate in LPS perception (Hussan et al., 2020). Previous research has shown that LysM receptor protein CEBiP and LysM receptor kinase CERK1 could form a chitin-enhanced heteromeric complex to activate PTI signal transduction in rice (Kaku et al., 2006; Shimizu et al., 2010). Recognition of chitin in *Arabidopsis* is mediated by CERK1, LYK4, and LYK5 through homodimerization and phosphorylation (Miya et al., 2007; Cao et al., 2014). Atlyk4/Atlyk5-2 double mutants resulted in a complete loss of chitin-triggered immunity, including ROS accumulation and resistance to *A. brassicicola* (Cao et al., 2014). Recent research indicated that LYK2 could constitutively interact with LYK5 in the cell plasma membrane to activate pathogen-elicitor-induced resistance (Giovannoni et al., 2021). In addition, LYK2 contributes to both basal and elicitor-triggered resistance to bacteria, and overexpression of *LYK2* enhances resistance to *P. syringae* and increases the expression of resistant-related genes during pathogen infection. Paparella et al. (2014) reported that the functional absence of *AtLYK3* could affect SA-mediated responses. In addition to chitin recognition, CERK1 and two LysM domain proteins (LYP2, LYP3) are indispensable for peptidoglycan perception and immunity to bacterial pathogen infection (Willmann et al., 2011). Hussan et al. (2020) reported that an LRR-RLK protein LIK1 is involved in *Xcc* LPS perception, then phosphorylated by CERK1. The above suggests that the recognition of *Xcc* by the receptors plays an important role in the cabbage’s defense response to *Xcc* infection.

Plants recognize pathogen PAMPs through PRR receptors and co-receptors on the cell membrane surface and phosphorylate BIK1. Activation of BIK1 inhibits pathogen growth by positively regulating ROS burst through phosphorylation of RbohD (Yuan et al., 2021). Previous research has shown that BIK1 paralog proteins PBL1, PBL2, and PBS1 are involved in the regulation of PTI. The *pbl* mutant is compromised in several PTI responses triggered by PAMPs (Lu et al., 2010; Zhang J. et al., 2010). In our study, *BIK1* and most *PBS1*-like genes were upregulated in *Xcc*-infected samples of the R line compared with the *Xcc*-infected samples of the S line. Moreover, a DENN domain protein gene, *SCD1*, was found to be downregulated in *Xcc*-infected samples of the R line compared with the *Xcc*-infected samples of the S line. Korasick et al. (2010) identified that *SCD1* was a negative regulator of basal resistance against bacteria, and an *scd1-1* mutant exhibits constitutive activation of defense responses. Desclos-Theveniau et al. (2012) reported that *LecRK-V.5* negatively regulates stomata innate immunity and overexpression lines demonstrate defective stomata closure, leading to pathogenic bacteria entering the leaves and serious disease. Moreover, the reduction of *LecRK-V.5* expression increases SA and ROS accumulation causes insensitivity to COR-mediated stomata re-opening and suppresses JA-induced defense responses (Gilardoni et al., 2011; Arnaud et al., 2012; Desclos-Theveniau et al., 2012). In our study, two lectin receptor kinase genes, *LecRK-V.5*, were significantly downregulated in *Xcc*-infected samples of the R line compared with the *Xcc*-infected samples of the S line (**Figure 9**, **Supplementary Table 6**), supporting the important roles of the *LecRK-V.5* gene in the interaction between cabbage and *Xcc*. Taken together, our analysis suggested that the R line could more actively mobilize these transmembrane signaling receptors or co-receptors to sense *Xcc* than the S line to activate the cabbages’ defense responses to *Xcc* infection (see Step 1 in the model, **Figure 9**).

### Responses of cabbage to pathogen entry and colonization during *X. campestris* infection

Plants have evolved defense mechanisms for stomata closure and/or inhibiting pathogen-mediated stomata re-open upon sensing PAMPs to actively prevent bacteria from entering plant leaves (Melotto et al., 2006). Stomata operate as osmotic machines when the plasma membrane H^+^-ATPase is activated. The activity of this proton pump induces H^+^ extrusion, causes membrane potential hyperpolarization, induces K^+^_in_ channels while deactivating K^+^_out_ channels, and uptakes charged solutes and water, leading to the guard cells to swell and open (Schroeder and Neher, 1987). Several pathogens can re-open stomata by hijacking these regulatory elements (Liu et al., 2009). Interestingly, three H^+^-ATPase genes were downregulated in *Xcc*-infected samples of the R line compared with the *Xcc*-infected samples of the S line. This indicated that the R line could more actively promote stomata closure and/or inhibit pathogen-mediated stomata re-open than the S line to protect against *Xcc* in the plant leaf (see Step 2 in the model, **Figure 9**, **Supplementary Table 6**). However, two H^+^-ATPase genes, *AHA1* and *AHA2*, were upregulated at 7 dpi and 12 dpi, respectively. This may suggest that the R line allows the stomata to absorb more CO_2_ for photosynthesis at the late infection stage to maintain the energy for growth and resistance. In addition to H^+^ ions, the transport of Ca^2+^ ions is also involved in the regulation of immune-associated stomata movement. Four *CAXs* genes were downregulated in *Xcc*-infected samples of the R line compared with the *Xcc*-infected samples of the S line, which might block the efflux of Ca^2+^ ions from the cytosol (**Figure 9**, **Supplementary Table 6**). Research has indicated that the deletion of *CAX* genes results in ROS and SA accumulation, thereby increasing disease resistance (Modareszadeh et al., 2020; Zhang et al., 2020). Elevation of cytosolic Ca^2+^ ions is known to repress H^+^-ATPase activity, cause apoplastic alkalinization, activate K^+^_out_ channels, and lead to stomatal closure (Bolwell et al., 2002).

Apoplastic alkalinization has been shown to be important for the generation of ROS by cell wall-associated peroxidases in response to pathogen attacks (Bolwell et al., 2002). A previous study has shown that repressing the cell wall peroxidase gene *PRX34* could impair the oxidative burst and cause enhanced susceptibility to both bacterial and fungal pathogens (Daudi et al., 2012). The GLPs provide resistance to H_2_O_2_ accumulation (Christensen et al., 2004). Moreover, hypoxia-response unknown protein (HUP) genes have been found to be substrates of stress-related MAPKs, acting as positive regulators of plant immune responses, and overexpression of *HUP* leads to ROS accumulation and enhanced disease resistance (Palm-Forster et al., 2017). In our study, these genes were also upregulated in *Xcc*-infected samples of the R line compared with the *Xcc*-infected samples of the S line. The accumulation of ROS in cabbage may increase resistance to *Xcc*. However, excessive ROS accumulation can cause biofilm system damage and even cell death (Lushchak, 2011). To adapt to ROS toxicity, plants usually protect themselves against ROS by having an antioxidant system that maintains ROS concentrations. In our data, two catalase genes (*CAT2*, *CAT3*) and two ascorbate peroxidase genes (*APX2*, *APX5*) were upregulated in *Xcc*-infected samples of the R line compared with the *Xcc*-infected samples of the S line, and they encode enzymes that scavenge hydrogen peroxide in plants (**Figure 9**, **Supplementary Table 6**). We also found several antioxidant DAMs upregulated in *Xcc*-infected samples of the R line compared with the *Xcc*-infected samples of the S line, including proline, ascorbic acid, and glutathione (**Figure 9**, **Supplementary Table 2**). Moreover, compared with the S line’s control, these metabolites are downregulated in the S line. Similar results were observed in a temporal and spatial assessment of the interaction between cabbage and *Xcc* (Gay and Tuzun, 2000).

Based on our data, during *Xcc* infection, the R line may effectively sense pathogens, prevent pathogens from entering leaves by regulating stomata closure, and activate oxygen bursts. In addition, while efficiently controlling pathogens, the R line could maintain the balance of oxidative stress in cabbage by coordinating the above genes and metabolites to prevent damage to plants due to the excessive plant defense responses (see Step 3 in the model, **Figure 9**).

### Defense responses of cabbage induced by *X. campestris* virulence effectors

To overcome plant basal defense responses, pathogens often secrete virulence-effector proteins into the host cell to subvert immunity and promote pathogenesis (Jones and Dangl, 2006). As a result of host–pathogen coevolution, plant resistance genes can activate their own defense responses by recognizing the perturbation of plant target proteins by pathogenic effectors (Zhang et al., 2017). The NLR protein SUMM2 was shown to indirectly recognize the disruption of the MEKK1–MKK1/2–MPK4 kinase cascade by pathogen effectors through CRCK3, which activates SUMM2‐mediated defense responses (Zhang et al., 2017; Devendrakumar et al., 2018). Our current study showed that *CRCKs* and most of *MEKK1* were downregulated in the R line compared with the S line (**Figure 9**, **Supplementary Table 6**). This may also indicate that this disease-resistant model or similar model also exists in cabbage and *Xcc* interactions. Previous research has shown the *Xcc* effector AvrAC uridylylates BIK1 to reduce its kinase activity and consequently inhibit downstream immunity (Feng et al., 2012). In plants, Wang et al. (2015) reported that BIK1 paralog PBL2 acts as a decoy for AvrAC enzymatic activity, and the plant immune receptor ZAR1 can indirectly recognize uridylylation of PBL2 by AvrAC through RKS1/ZRK1. ZAR1 has been shown to form pre-activation complexes with ZRK3, ZRK6, and ZRK15 (Wang et al., 2015; Seto et al., 2017). In our data, *ZAR1* and *ZRK4* were upregulated at all three-time points in *Xcc*-infected samples of the R line compared with the *Xcc*-infected samples of the S line (**Figure 9**, **Supplementary Table 6**). Notably, ZAR1 has been shown to form complexes with the protein kinase ZED1 to recognize the effector HopZ1a through HopZ1a-mediated acetylation of ZED1 (Lewis et al., 2013). *ZED1* was upregulated in *Xcc*-infected samples of the R line compared with the *Xcc*-infected samples of the S line. Based on our data, ZAR1, ZED1, and ZRK4 may be involved in the recognition of *Xcc* virulence effectors, thereby activating the downstream immune responses (see Step 4 in the model, **Figure 9**).

The type III effector AvrRpt2 can cleave RIN4 and lead to the activation of RPS2-mediated disease resistance (Mackey et al., 2003). RIN4 can also interact with RPM1, and manipulation of RIN4 by another effector, AvrRpm1, could trigger the activation of defenses induced by RPM1 (Mackey et al., 2002). AvrRpt2 can inhibit RPM1-mediated disease resistance when *Arabidopsis* interacts with *Pst DC3000* expressing both avrRpm1 and avrRpt2 (Ritter and Dangl, 1996). Interestingly, Mackey et al. (2003) reported that overexpression of *RIN4* can suppress the ability of *RPS2*-mediated disease resistance and the interference of AvrRpt2 on RPM1 function. This partly supports our study. In our data, *RIN4* and *RPM1* were upregulated in *Xcc*-infected samples of the R line compared with the *Xcc*-infected samples of the S line, and no differential expression of *RPS2* was detected between the two lines (**Figure 9**, **Supplementary Table 6**). Therefore, the resistance to *Xcc* effector activation in cabbage may depend on RPM1. Earlier studies showed that the *Xcc* bacterium carries effector AvrXccC, highly homologous to effector AvrB of *P. syringae*, whose activities lead to enhanced defense responses in the plants (Mackey et al., 2002; White et al., 2009).

Previous studies also found that cell autophagy plays an important role in plant immune responses. Feng (2012) reported that cell autophagy negatively regulates AvrAC-activated ETI responses in host plants. They also found that AvrAC has the ability to interact with ATG8a of the autophagy pathway. In addition, the resistance activated by AvrAC on *atg5* and *atg7* mutants was further expanded, resulting in a decrease in *Xcc* growth in mutants compared to wild-type plants. Similar results have been found in our data (**Figure 9**, **Supplementary Table 6**). Seven autophagy genes were downregulated in *Xcc*-infected samples of the R line compared with the *Xcc*-infected samples of the S line, including *ATG3*, *ATG7*, *ATG8a*, two *ATG10*, and *ATG101*, suggesting cell autophagy might be involved in the resistance of the R line to *Xcc*.

### Reprogramming of hormone metabolism pathways in resistance to *X. campestris*

Plant hormone resistance pathways are mainly dependent on salicylic acid, jasmonic acid, and ethylene, which are involved in the regulation of different types of disease resistance signaling pathways (Bari and Jones, 2009). To study whether hormone-mediated pathways in cabbage against *Xcc*, we examined the profile of DEGs and DAMs associated with plant hormones. We found that many, especially SA, JA, and ET, played crucial roles in the interaction of cabbage following *Xcc* infection (see Step 5 in the model, **Figure 9**, **Supplementary Tables 2**, **6**). The two enzymes, phenylalanine ammonia-lyase 1 (PAL1) and isochorismate synthase 1 (ICS1), are the key enzymes in the SA synthesis pathway. The two enzymes of salicylic acid glucosyltransferase 1 (SGT1) and BA/SA carboxyl methyltransferase 1 (BSMT1) play important roles in the SA modification pathway, catalyzing the formation of SA 2-O-β-D-glucoside (SAG) and methyl salicylate (MeSA), respectively. SAG and MeSA do not appear to be biologically active and are incapable of inducing SA-mediated defense responses by themselves (Hennig et al., 1993; Dempsey et al., 2011). Our data showed that two *ICS1* and two *PAL1* genes were upregulated in *Xcc*-infected samples of the R line compared with the *Xcc*-infected samples of the S line, while *SGT1* and *BSMT1* were downregulated. Based on our metabolome data, the accumulation of SA in *Xcc*-infected samples of the R line was higher than that in *Xcc*-infected samples of the S line. Moreover, SAG was downregulated in *Xcc*-infected samples of the R line compared with the *Xcc*-infected samples of the S line. Compared with the S line’s control, SAG was upregulated in *Xcc*-infected samples of the S line. Early research indicated that decreased resistance by AtSGT1 overexpression results from a reduction in SA content, which is at least partly due to the increase in MeSAG and MeSA levels at the expense of SA (Song et al., 2008). For transcriptional regulation of the SA-mediated pathways in cabbage responsive to *Xcc* infection, we found that *SARD1* and *MYB96* were upregulated in *Xcc*-infected samples of the R line compared with the *Xcc*-infected samples of the S line. Previous studies have reported that overexpression of *SARD1* can control SA synthesis by regulating *ICS1* at the transcriptional level, thereby activating defense responses (Zhang Y. X. et al., 2010). Moreover, *MYB96* mediates upregulated expression of *ICS1* and *PAL1* by promoting SA biosynthesis to induce pathogen resistance response (Seo and Park, 2010). Two other transcription factors, *MYB70* and *WHY1*, were established as important downstream components of SA-mediated defense responses, which are upregulated in *Xcc*-infected samples of the R line compared with the *Xcc*-infected samples of the S line. *WRKY70* acts as the activator of SA-induced genes and the inhibitor of JA reaction genes, integrating JA- and SA-mediated signal convergence nodes in plant defense (Desveaux et al., 2004; Li et al., 2004). Compared with *Xcc*-infected samples of the S line, five JA-inducible genes (four coronatine-induced genes and one vegetative storage protein gene) were significantly downregulated in *Xcc*-infected samples of the R line after inoculation with *Xcc*. A recent study reported that SA activates the RpfB-dependent diffusible signaling factor turnover by altering the cytoplasmic pH in *Xcc*, thereby negatively regulating the production of virulence factors (Song et al., 2022). We also found *NDR1*, *EDS1*, and its interaction partner *PAD4* were upregulated in *Xcc*-infected samples of the R line compared with the *Xcc*-infected samples of the S line and act as regulators of signaling to mediate the accumulation of SA, which activates systemic acquired immunity (SAR) in plants (Dempsey et al., 2011). Rong et al. (2010) demonstrated that NDR1 is required for *Xcc*‐induced defense responses in Arabidopsis. Combined with our data, this suggests that the SA signaling pathway plays an important role in cabbage resistance to *Xcc* infection.

The SA and JA/ET defense pathways are mutually antagonistic. Therefore, we evaluated the regulation pattern of JA metabolism in *Xcc*-infected samples of the R line compared with the *Xcc*-infected samples of the S line (**Figure 9**, **Supplementary Tables 2**, **6**). Our data showed that two metabolites 13(S)-hydroperoxy-octadecatrienoic acid and hexadecanedioic acid were downregulated at 12 dpi, which belongs to the upstream of the JA synthesis pathway. Another upstream metabolite 12-oxo-phytodienoic acid, was upregulated at 7 dpi and downregulated at 12 dpi. The five enzymes, lipoxygenase, OPDA reductase, acyl-CoA oxidase, a multifunctional protein, and 3-ketoacyl-CoA thiolase are the key enzymes at the upstream of the JA synthesis pathway (Acosta and Farmer, 2010). In our data, these genes were downregulated in *Xcc*-infected samples of the R line compared with the *Xcc*-infected samples of the S line, which might result in the reduction of final catalytic products. Constantino et al. (2013) reported that *LOX3* negatively regulates induced systemic resistance to *C. graminicola* in maize. We found two *JAR1* genes, which catalyze the formation of biologically active jasmonyl-isoleucine (JA-Ile) conjugates, were downregulated in *Xcc*-infected samples of the R line compared with the *Xcc*-infected samples of the S line (Staswick and Tiryaki, 2004). JA-Ile is a negative regulator of stomatal defense by specifically promoting interaction between JAZ1 and COI1 (Melotto et al., 2006). We found *JAZ1* and *COI1* were downregulated in *Xcc*-infected samples of the R line compared with the *Xcc*-infected samples of the S line. As a molecular mimic of JA-Ile, COR can also manipulate JAZ-COI1 co-receptor complexes, leading to the removal of JAZ repressors and liberating MYC2 from inhibition. Therefore, COR activation of JA signaling induces the expression of *ANAC019*, *ANAC055*, and *ANAC072* directly through *MYC2* (Zheng et al., 2012; Kazan and Manners, 2013). These NAC TFs inhibit the accumulation of SA by repressing ICS1 and activating BSMT1, leading to the virulence effects of COR in plants to subvert immunity and promote pathogenesis. Interestingly, *MYC2*, *MYC3*, *MYC4*, *ANAC019*, and *ANAC072* were downregulated in *Xcc*-infected samples of the R line compared with the *Xcc*-infected samples of the S line. Moreover, MYC2 can specifically suppress *EDS1* promoter activity to negatively regulate the SA signaling pathway (Qiu, 2016). Based on our data, an induced SA signal pathway and a repressed JA signal pathway were proposed in the R line during *Xcc* infection.

Synergistic interactions also exist between the SA and JA/ET defense pathways. According to transcriptome and metabolome analyses, we evaluated the regulation pattern of ET metabolism in *Xcc*-infected samples of the R line compared with the *Xcc*-infected samples of the S line (**Figure 9**, **Supplementary Tables 2**, **6**). We found 1-aminocyclopropane-1-carboxylic acid (ACC) was downregulated in *Xcc*-infected samples of the R line compared with the *Xcc*-infected samples of the S line. The enzymes that catalyze AdoMet to ACC and ACC to ethylene are ACC synthase (ACS) and ACC oxidase (ACO), respectively (Bleecker and Kende, 2000). We found *ACO* genes were upregulated in *Xcc*-infected samples of the R line compared with the *Xcc*-infected samples of the S line. Moreover, we found that *EIN3* and two *EIL1* genes were upregulated in *Xcc*-infected samples of the R line compared with the *Xcc*-infected samples of the S line. As the core TFs, EIN3/EIL1 activates a large number of downstream pathways. For example, ROS production is diminished in *ein3 eil1* double mutants under the Flg22 treatment (Mersmann et al., 2010). In rice, EIL1 is involved in disease resistance by activation of ROS during *M. oryzae* infection (Yang et al., 2017). However, Zhang et al. (2016) reported that LeEILs could activate the expression of TERF1 and play a role in ROS scavenging in tobacco. These data suggest that ET mediates SA-induced ROS production in an EIN3/EIL1-dependent manner. Moreover, EIN3/EIL1 can negatively regulate ICS1 expression and SA biosynthesis to suppress plant immunity (Chen et al., 2009). Therefore, EIN3/EIL1 may play an important role in cabbage during *Xcc* infection by participating in the crosstalk of ET and SA. In addition, we found three *ERF4* and one *ERF11* gene were upregulated in *Xcc*-infected samples of the R line compared with the *Xcc*-infected samples of the S line. AtERF4 and AtERF11, both belonging to the VIIIa (B-1a) subgroup of the ERF family, are reported to regulate defense responses to pathogens by the SA/ET-regulated signaling pathways (Nasir et al., 2005; Wu et al., 2020). Lai et al. (2013) reported that overexpression of *BrERF11* enhanced resistance to *R. solanacearum* infection, and the transcript levels of *BrERF11* were significantly increased by exogenous SA. They also found a significant increase in the transcription of the ACC oxidase gene and the production of H_2_O_2_ in the *ERF11* overexpression transgenic lines, while the transcription of *CAT1*, involved in scavenging H_2_O_2_ production, was decreased. A recent study found that ERF11 could activate the transcription of *BT4* to increase resistance to *P. syringae* (Zheng et al., 2019). They also found that BT4 functions in the defense process against pathogens and is modulated by the SA/ET signaling pathway. In our study, we found one *BT4* and two *BT5* genes were upregulated in *Xcc*-infected samples of the R line compared with the *Xcc*-infected samples of the S line. Unexpectedly, another *BT4* gene was downregulated. Overall, we suggest the ethylene signaling pathway acts as a regulatory node to control the cabbage immune responses to *Xcc*, which not only promotes SA-mediated immune responses but also prevents unregulated defense responses that are detrimental to plant growth and development.

Previous studies showed that the effectors of *Xcc* mediate the manipulation of ABA biosynthetic pathways as a virulence mechanism, leading to the suppression of defense responses (Ho et al., 2013). Moreover, Hu et al. (2022) reported that phytopathogen effectors bind and inhibit the function of TOPPs by negatively regulating SnRK2 phosphorylation and hijacking ABA signaling to induce water soaking in plant leaves, which causes the apoplast to become a water-rich environment conducive to the growth of pathogens. Another study found that *Xcc* infection increases the water percentage of *B. oleracea* seedlings (Vega-alvarez et al., 2021). In our data, we found *TOPP2* was upregulated and three *SnRKs* were downregulated in *Xcc*-infected samples of the R line compared with the *Xcc*-infected samples of the S line. In addition, ABA and NCED9 of the ABA biosynthesis pathway were downregulated at 12 dpi in *Xcc*-infected samples of the R line compared with the *Xcc*-infected samples of the S line (**Figure 9**, **Supplementary Table 6**). Moreover, ABA was upregulated in *Xcc*-infected samples of the S line compared with the control samples of the S line (**Figure 9**, **Supplementary Table 2**). These results were similar to a proteome analysis of the interaction between cabbage and *Xcc* (Santos et al., 2019). Based on our data, the R line may enhance plant disease responses to *Xcc* by reducing the accumulation of ABA and suppressing ABA signaling. This will inhibit the colonization of pathogens.

### Sugar metabolisms pathway are associated with resistance to *X. campestris*

Plants also actively compete with pathogens for nutrients, thus blocking pathogens’ access to sugar to control diseases (Oliva and Quibod, 2017). Zhang et al. (2018) reported that glucose as a carbon source and diffusible signal factor family signal precursor is a benefit to *Xcc* for virulence factor production and pathogenicity. A previous study showed that stomatal opening is impaired in plant guard cells lacking STP transporters (Flütsch et al., 2020). SUCs are involved in sucrose loading into the phloem as an important transporter, and sucrose is released from phloem parenchyma cells into the apoplast by members of the SWEET transporter family (Chen et al., 2012; Ludewig and Flugge, 2013). Most of these genes were downregulated in *Xcc*-infected samples of the R line compared with the *Xcc*-infected samples of the S line, which suggests that the R line might more actively regulate sugar transport to protect against *Xcc* infection. Bacterial pathogen effector molecules are able to increase the activity of SWEET systems to enhance sugar efflux (Chen et al., 2012; Zhang et al., 2018). Furthermore, we also found some of these genes were upregulated in *Xcc*-infected samples of the R line compared with the *Xcc*-infected samples of the S line, which enhances plants’ monosaccharide uptake activity to compete with pathogens for extracellular sugars (Yamada et al., 2016).

During infection, pathogens modify the plant sugar metabolism for the growth of plant pathogens, competing for valuable energy for plants’ normal growth and defense responses (Tauzin and Giardina, 2014). Zhang et al. (2018) reported that some sucrose hydrolase genes are upregulated in both pathogens and plants during *Xcc* infection. Zhao et al. (2015) found that elevated CWINVs activity contributes to callose deposition and cell wall reinforcement during pathogen infection, therefore, partially induces pathogen resistance. Xiang et al. (2011) reported that A/N-INVs are involved in the dynamic balance of oxidative stress by regulating the expression of oxidative stress defense genes. In our data, these invertase genes were differentially expressed in *Xcc* infected samples of the R line compared with the *Xcc*-infected samples of the S line. In addition, *IDD2*, similar to maize indeterminate1 (*ID1*), was downregulated in *Xcc*-infected samples of the R line compared with the *Xcc*-infected samples of the S line. *id1* mutant showed increased accumulation of sucrose and more susceptibility to *Ustilago maydis* (Kretschmer et al., 2017). We also found two starch synthase genes were upregulated and *BAM5* was downregulated in *Xcc*-infected samples of the R line compared with the *Xcc*-infected samples of the S line. Rapid starch degradation in guard cells has been proven to play an important role in stomata opening (Horrer et al., 2016). Moreover, we found malic acid, citric acid, isocitric acid, succinic acid, and fumaric acid were upregulated in *Xcc*-infected samples of the R line compared with the *Xcc*-infected samples of the S line (**Supplementary Table 2**). Based on the above data, the R line may actively regulate sugar production and restrict mobilization into the apoplast to block *Xcc*’s access to nutrients as an alternative way to prevent colonization. In summary, cabbage and *Xcc* engage in a tug-of-war in which the cabbage limits *Xcc*’s access to nutrients as an effective defense strategy (see Step 6 in the model, **Figure 9**).

### Phenylpropanoid and flavonoid metabolisms pathway are associated with resistance to *X. campestris*

Phenylpropanoids, flavonoids, and their derivatives can function as important components of defense responses in plant–pathogen interactions (Ranjan et al., 2019; Li et al., 2021). In our study, we noted the differential regulation of transcripts and metabolites related to the phenylpropanoid and flavonoid pathways between the R and S lines (**Supplementary Tables 2**, **6**). At the transcript level, we observed upregulation of genes encoding the enzymes *PAL*, chalcone synthase (*CHS*), chalcone isomerase-like (*CHIL*), flavonoid 3′-monooxygenase (*F3′M*), and flavonol synthase (*FLS*) in *Xcc*-infected samples of the R line compared with the *Xcc*-infected samples of the S line. Moreover, our data identified lower expression of genes encoding the enzyme coumarate 3-hydroxylase (*C3H*) in *Xcc*-infected samples of the R line compared with the *Xcc*-infected samples of the S line, while half of the annotated 4-coumarate: CoA ligase (*4CL*) and cinnamic acid 4-hydroxylase (*C4H*) genes were upregulated in *Xcc*-infected samples of the R line compared with the *Xcc*-infected samples of the S line. Changes in the expression levels of these genes might eventually result in a consistent accumulation of the products of the catalytic reactions.

In accordance with the metabolome data, we observed different accumulations of the metabolites (or their derivatives), including cinnamic acid, p-coumaric acid, p-coumaroylquinic acid, Caffeoyl quinic acid, p-coumaroylshikimic acid, caffeic acid, chlorogenic acid, ferulic acid, coniferin, sinapic acid, syringin, naringenin, prunin, luteolin, and kaempferol in *Xcc*-infected samples of the R line compared with the *Xcc*-infected samples of the S line (**Supplementary Table 2**). Most of these metabolites were upregulated. Islam et al. (2018) reported that flavonoids positively regulate the resistance of Chinese cabbage to *Xcc*. Moreover, we found that two flavonoid pathway metabolites, chlorogenic acid and caffeic acid, can inhibit the growth of *Xcc* in the culture medium (with or without agar, **Figure 8**). These two metabolites were all upregulated in *Xcc*-infected samples of the R line compared with the *Xcc*-infected samples of the S line. It can be speculated that the antibacterial function of flavonoid pathway metabolisms contributed to the cabbage’s resistance to *Xcc*. Based on our data, we propose that reprogramming of the phenylpropanoid and flavonoid metabolism leads to resistance of the R line to *Xcc*.

## Conclusions

In summary, we observed that the transcript and metabolite profiles between the R and S lines undergo marked reprogramming during *Xcc* infection. A large number of pathogen-related genes/metabolites were identified between the R and S lines, and therefore, we proposed a working model of the cabbage–*Xcc* interactions (**Figure 9**, **Supplementary Tables 2**, **6**). This model helps to better understand the defense response of cabbage to *Xcc* infection and provides research clues and resource data.

The initial mechanism of plant defense signals occurs at the beginning of infection, with the PAMPs being detected by the PRR proteins of the cabbage cell. Between the resistant and susceptible cabbage lines, we found several genes that were upregulated and probably involved in these perception pathways, including *BIK1*, *PBLs*, *LYP1/2*, *LYKs*, and *SOBIR1*. Some other genes were downregulated and might play negative roles in these pathways. These transmembrane signaling receptors, or co-receptors, can be essential to sense *Xcc* and promote plant innate immunity. After perception, we found the R line may more actively promote stomatal closure and/or inhibit pathogen-mediated stomatal re-open than the S line by preventing *Xcc* from entering leaves. Genes involved with ROS production and scavenging were all upregulated, including *PRX34*, *GLPs*, *CAT2/3*, and *APX2/5*. Moreover, several antioxidant metabolites were upregulated in *Xcc*-infected samples of the R line compared with the *Xcc*-infected samples of the S line. This may indicate that a balance of ROS accumulation, maintaining some genes/metabolites upregulated and others downregulated, may be important for effectively inhibiting or reducing *Xcc* survival and avoiding damage to plant growth.

Once the pathogen overcomes the first line of defense, successful pathogens often secrete virulence-effector proteins into the host cell to subvert immunity and promote pathogenesis. At this stage, plants initiate another mechanism to interact with the pathogen effectors. In this study, we identified upregulated genes *ZED1*, *ZRKs*, *ZAR1*, *RIN4*, and *RPM1*, which have been reported as the receptors for effectors’ perception. Moreover, compared with the *Xcc*-infected samples of the S line, cell autophagy genes were shown to be downregulated in *Xcc*-infected samples of the R line, which may indicate a negative regulation of this pathway. Plant defense responses are highly correlated with the levels of plant hormones, especially SA. As seen in the model, the SA pathway was activated while the JA pathway was inhibited in the resistant cabbage line during *Xcc* infection. In this study, SA plays a positive role in the induction of defense responses against *Xcc* in cabbage. ET can modulate the balance of defense responses stimulated by SA, while JA and ABA negatively regulate resistance to *Xcc*. Moreover, cabbage protects itself from *Xcc* by regulating energy metabolism. Secondary metabolites also play an important role in plant defense. Several phenylpropanoid and flavonoid-associated metabolites were identified with increased abundance and were associated with plant defense responses. Moreover, two flavonoid pathway metabolites, chlorogenic acid and caffeic acid, can significantly inhibit the proliferation of *Xcc*. Our present results provide important guidance for a better understanding of the molecular resistance of *Xcc*, which may aid in promoting the breeding of resistant varieties to control the black rot of cabbage.

## Data availability statement

The data presented in the study are deposited in the National Genomics Data Center repository, accessionnumber CRA007496.

## Author contributions

QS performed the experiments, analyzed the data, and wrote the draft of the manuscript. QZ contributed to the acquisition of the data. WH, DL, LC, and QZ contributed to the preparation of the manuscript. EZ, ZX, and BL obtained funding for the project and contributed to the preparation of the manuscript. QS and BL revised and finalized the manuscript. All authors contributed to the article and approved the submitted version.

## Funding

This work was supported by the National Key Research and Development Program of China (2017YFD0101804) to EZ, the National Technical System of Bulk Vegetable Industry (CARS-23-G22) to ZX, and the National Natural Science Foundation of China (32070333) to BL.

## Conflict of interest

The authors declare that the research was conducted in the absence of any commercial or financial relationships that could be construed as a potential conflict of interest.

## Publisher’s note

All claims expressed in this article are solely those of the authors and do not necessarily represent those of their affiliated organizations, or those of the publisher, the editors and the reviewers. Any product that may be evaluated in this article, or claim that may be made by its manufacturer, is not guaranteed or endorsed by the publisher.

## Supplementary material

Supplementary Figure 1 Correlation analysis of the variance-stabilized estimated raw counts of differentially accumulated metabolites.

Supplementary Figure 2 Heatmap visualization of identified metabolites in comparisons. **(A)**: R7M-CK *vs* R7M; **(B)**: R12M-CK *vs* R12M; **(C)**: S7M-CK *vs* S7M; **(D)**: S12M-CK *vs* S12M; **(E)**: S7M *vs* R7M; **(F)**: S12M *vs* R12M.

Supplementary Figure 3 Top 20 KEGG enrichment pathway of identified DAMs in comparisons. **(A)**: S7M *vs* R7M; **(B)**: S12M *vs* R12M.

Supplementary Figure 4 GO assignment of identified DEGs in comparisons. **(A)**: Comparison between control and inoculated resistant line. **(B)**: Comparison between control and inoculated susceptible line. **(C)**: Comparison between inoculated resistant and susceptible line.

Supplementary Figure 5 Top 50 enriched KEGG pathways at all nine comparisons. **(A)**: R2T-CK *vs* R2T; **(B)**: R7T-CK *vs* R7T; **(C)**: R12T-CK *vs* R12T; **(D)**: S2T-CK *vs* S2T; **(E)**: S7T-CK *vs* S7T; **(F)**: S12T-CK *vs* S12T; **(G)**: S2T *vs* R2T; **(H)**: S7T *vs* R7T; **(I)**: S12T *vs* R12T.

Supplementary Figure 6 Confirmation of expression profiles of select DEGs in R line compared with S line using qRT-PCR.

## References

[B1] Abdelrahman M. Nakabayashi R. Mori T. Ikeuchi T. Mori M. Murakami K.. (2020). Comparative metabolome and transcriptome analyses of susceptible asparagus officinalis and resistant wild a. kiusianus reveal insights into stem blight disease resistance. Plant Cell Physiol. 61, 1464–1476. doi: 10.1093/pcp/pcaa054 32374863

[B2] Acosta I. F. Farmer E. E. (2010). “Jasmonates,” in The arabidopsis book, ed. C. Somerville and E. Meyerowit (Rockville, Maryland: The American Society of Plant Biologists), vol. 8. doi: 10.1199/tab.0129 PMC3244945 22303255

[B3] Arnaud D. Desclos-Theveniau M. Zimmerli L. (2012). Disease resistance to pectobacterium carotovorum is negatively modulated by the arabidopsis lectin receptor kinase LecRK-V.5. Plant Signaling Behav. 7, 1070–1072. doi: 10.4161/psb.21013 PMC3489629 22899085

[B4] Bari R. Jones J. (2009). Role of plant hormones in plant defence responses. Plant Mol. Biol. 69, 473–488. doi: 10.1007/s11103-008-9435-0 19083153

[B5] Bleecker A. B. Kende H. (2000). Ethylene: A gaseous signal molecule in plants. Annu. Rev. Cell Dev. Bi. 16, 1–18. doi: 10.1146/annurev.cellbio.16.1.1 11031228

[B6] Bolwell G. P. Bindschedler L. V. Blee K. A. Butt V. S. Davies D. R. Gardner S. L.. (2002). The apoplastic oxidative burst in response to biotic stress in plants: A three-component system. J. Exp. Bot. 53, 1367–1376. doi: 10.1093/jexbot/53.372.1367 11997382

[B7] Cao Y. Liang Y. Tanaka K. Nguyen C. T. Jedrzejczak R. P. Joachimiak A.. (2014). The kinase LYK5 is a major chitin receptor in arabidopsis and forms a chitin-induced complex with related kinase CERK1. Elife 3, e3766. doi: 10.7554/eLife.03766 PMC4356144 25340959

[B8] Chen L. Q. Qu X. Q. Hou B. H. Sosso D. Osorio S. Fernie A. R.. (2012). Sucrose efflux mediated by SWEET proteins as a key step for phloem transport. Science 335, 207–211. doi: 10.1126/science.1213351 22157085

[B9] Chen H. M. Xue L. Chintamanani S. Germain H. Lin H. Q. Cui H. T.. (2009). ETHYLENE INSENSITIVE3 and ETHYLENE INSENSITIVE3-LIKE1 repress SALICYLIC ACID INDUCTION DEFICIENT2 expression to negatively regulate plant innate immunity in arabidopsis. Plant Cell 21, 2527–2540. doi: 10.1105/tpc.108.065193 19717619 PMC2751940

[B10] Christensen A. B. Thordal-Christensen H. Zimmermann G. Gjetting T. Lyngkjaer M. F. Dudler R.. (2004). The germinlike protein GLP4 exhibits superoxide dismutase activity and is an important component of quantitative resistance in wheat and barley. Mol. Plant Microbe 17, 109–117. doi: 10.1094/MPMI.2004.17.1.109 14714874

[B11] Constantino N. N. Mastouri F. Damarwinasis R. Borrego E. J. Moran-Diez M. E. Kenerley C. M.. (2013). Root-expressed maize lipoxygenase 3 negatively regulates induced systemic resistance to colletotrichum graminicola in shoots. Front. Plant Sci. 4, 510. doi: 10.3389/fpls.2013.00510 PMC3867115 24391653

[B12] Daudi A. Cheng Z. Y. O'Brien J. A. Mammarella N. Khan S. Ausubel F. M.. (2012). The apoplastic oxidative burst peroxidase in arabidopsis is a major component of pattern-triggered immunity. Plant Cell 24, 275–287. doi: 10.1105/tpc.111.093039 22247251 PMC3289579

[B13] Dempsey D. A. Vlot A. C. Wildermuth M. C. Klessig D. F. (2011). “Salicylic acid biosynthesis and metabolism”. in The Arabidopsis book, ed. C. Somerville and E. Meyerowit (Rockville, Maryland: The American Society of Plant Biologists), vol. 9. doi: 10.1199/tab.0156 PMC3268552 22303280

[B14] Desclos-Theveniau M. Arnaud D. Huang T. Y. Lin G. Chen W. Y. Lin Y. C.. (2012). The arabidopsis lectin receptor kinase LecRK-V.5 represses stomatal immunity induced by pseudomonas syringae pv. tomato DC3000. PLos Pathog. 8, e1002513. doi: 10.1371/journal.ppat.1002513 22346749 PMC3276567

[B15] Desveaux D. Subramaniam R. Despres C. Mess J. N. Levesque C. Fobert P. R.. (2004). A "whirly" transcription factor is required for salicylic acid-dependent disease resistance in arabidopsis. Dev. Cell 6, 229–240. doi: 10.1016/S1534-5807(04)00028-0 14960277

[B16] Devendrakumar K. T. Li X. Zhang Y. L. (2018). MAP kinase signalling: Interplays between plant PAMP- and effector-triggered immunity. Cell Mol. Life Sci. 75, 2981–2989. doi: 10.1007/s00018-018-2839-3 29789867 PMC11105241

[B17] Feng F. (2012). The molecular mechanism of xanthomonas type III effector AvrAC to regulate plant innate immunity ([PhD thesis]. (Beijing, China: Tsinghua University).

[B18] Feng F. Yang F. Rong W. Wu X. G. Zhang J. Chen S.. (2012). A xanthomonas uridine 5 '-monophosphate transferase inhibits plant immune kinases. Nature 485, 114–149. doi: 10.1038/nature10962 22504181

[B19] Flütsch S. Nigro A. Conci F. Fajkus J. Santelia D. (2020). Glucose uptake to guard cells *via* STP transporters provides carbon sources for stomatal opening and plant growth. EMBO Rep, 21 (8) :e479719. doi: 10.15252/embr.201949719 PMC7403697 32627357

[B20] Gao M. H. Wang X. Wang D. M. Xu F. Ding X. J. Zhang Z. B.. (2009). Regulation of cell death and innate immunity by two receptor-like kinases in arabidopsis. Cell Host Microbe 6, 34–44. doi: 10.1016/j.chom.2009.05.019 19616764

[B21] Gay P. A. Tuzun S. (2000). Temporal and spatial assessment of defense responses in resistant and susceptible cabbage varieties during infection with xanthomonas campestris pv. campestris. Physiol. Mol. Plant P 57, 201–210. doi: 10.1006/pmpp.2000.0299

[B22] Gilardoni P. A. Hettenhausen C. Baldwin I. T. Bonaventure G. (2011). Nicotiana attenuata LECTIN RECEPTOR KINASE1 suppresses the insect-mediated inhibition of induced defense responses during manduca sexta herbivory. Plant Cell 23, 3512–3532. doi: 10.1105/tpc.111.088229 21926334 PMC3203443

[B23] Giovannoni M. Lironi D. Marti L. Paparella C. Vecchi V. Gust A. A.. (2021). The arabidopsis thaliana LysM-containing receptor-like kinase 2 is required for elicitor-induced resistance to pathogens. Plant Cell Environ. 44, 3545–3562. doi: 10.1111/pce.14192 34558681 PMC9293440

[B24] Halter T. Imkampe J. Mazzotta S. Wierzba M. Postel S. Bucherl C.. (2014). The leucine-rich repeat receptor kinase BIR2 is a negative regulator of BAK1 in plant immunity. Curr. Biol. 24, 134–143. doi: 10.1016/j.cub.2013.11.047 24388849

[B25] Hennig J. Malamy J. Grynkiewicz G. Indulski J. Klessig D. F. (1993). Interconversion of the salicylic acid signal and its glucoside in tobacco. Plant J. 4, 593–600. doi: 10.1046/j.1365-313X.1993.04040593.x 8252063

[B26] Horrer D. Flutsch S. Pazmino D. Matthews J. Thalmann M. Nigro A.. (2016). Blue light induces a distinct starch degradation pathway in guard cells for stomatal opening. Curr. Biol. 26, 362–370. doi: 10.1016/j.cub.2015.12.036 26774787

[B27] Ho Y. P. Tan C. M. Li M. Y. Lin H. Deng W. L. Yang J. Y. (2013). The AvrB_AvrC domain of AvrXccC of xanthomonas campestris pv. campestris is required to elicit plant defense responses and manipulate ABA homeostasis. Mol. Plant Microbe 26, 419–430. doi: 10.1094/MPMI-06-12-0164-R 23252460

[B28] Hu Y. Ding Y. Cai B. Qin X. Wu J. Yuan M.. (2022). Bacterial effectors manipulate plant abscisic acid signaling for creation of an aqueous apoplast. Cell Host Microbe 30, 518–529. doi: 10.1016/j.chom.2022.02.002 35247331

[B29] Hussan R. H. Dubery I. A. Piater L. A. (2020). Identification of MAMP-responsive plasma membrane-associated proteins in arabidopsis thaliana following challenge with different LPS chemotypes from xanthomonas campestris. PATHOGENS 9 (10): 787. doi: 10.3390/pathogens9100787 PMC7650673 32992883

[B30] Islam M. T. Lee B. R. Das P. R. La V. H. Jung H. I. Kim T. H. (2018). Characterization of p-coumaric acid-induced soluble and cell wall-bound phenolic metabolites in relation to disease resistance to xanthomonas campestris pv. campestris in Chinese cabbage. Plant Physiol. Bioch. 125, 172–177. doi: 10.1016/j.plaphy.2018.02.012 29455090

[B31] Jones J. D. G. Dangl J. L. (2006). The plant immune system. Nature 444, 323–329. doi: 10.1038/nature05286 17108957

[B32] Kaku H. Nishizawa Y. Ishii-Minami N. Akimoto-Tomiyama C. Dohmae N. Takio K.. (2006). Plant cells recognize chitin fragments for defense signaling through a plasma membrane receptor. P Natl. Acad. Sci. U.S.A. 103, 11086–11091. doi: 10.1073/pnas.0508882103 PMC1636686 16829581

[B33] Kazan K. Manners J. M. (2013). MYC2: The master in action. Mol. Plant 6, 686–703. doi: 10.1093/mp/sss128 23142764

[B34] Korasick D. A. McMichael C. Walker K. A. Anderson J. C. Bednarek S. Y. Heese A. (2010). Novel functions of stomatal cytokinesis-defective 1 (SCD1) in innate immune responses against bacteria. J. Biol. Chem. 285, 23340–23348. doi: 10.1074/jbc.M109.090787 PMC2906326 20472560

[B35] Kretschmer M. Croll D. Kronstad J. W. (2017). Maize susceptibility to ustilago maydis is influenced by genetic and chemical perturbation of carbohydrate allocation. Mol. Plant Pathol. 18, 1222–1237. doi: 10.1111/mpp.12486 27564861 PMC6638311

[B36] Lai Y. Dang F. F. Lin J. Yu L. Shi Y. L. Xiao Y. H.. (2013). Overexpression of a Chinese cabbage BrERF11 transcription factor enhances disease resistance to ralstonia solanacearum in tobacco. Plant Physiol. Bioch. 62, 70–78. doi: 10.1016/j.plaphy.2012.10.010 23201563

[B37] Lewis J. D. Lee A. Hassan J. A. Wan J. Hurley B. Jhingree J. R.. (2013). The arabidopsis ZED1 pseudokinase is required for ZAR1-mediated immunity induced by the pseudomonas syringae type III effector HopZ1a. P Natl. Acad. Sci. U.S.A. 110, 18722–18727. doi: 10.1073/pnas.1315520110 PMC3831984 24170858

[B38] Li J. Brader G. Palva E. T. (2004). The WRKY70 transcription factor: A node of convergence for jasmonate-mediated and salicylate-mediated signals in plant defense. Plant Cell 16, 319–331. doi: 10.1105/tpc.016980 14742872 PMC341906

[B39] Li P. Q. Ruan Z. Fei Z. X. Yan J. J. Tang G. H. (2021). Integrated transcriptome and metabolome analysis revealed that flavonoid biosynthesis may dominate the resistance of zanthoxylum bungeanum against stem canker. J. Agr. Food Chem. 69, 6360–6378. doi: 10.1021/acs.jafc.1c00357 34043342

[B40] Liu J. Elmore J. M. Fuglsang A. T. Palmgren M. G. Staskawicz B. J. Coaker G. (2009). RIN4 functions with plasma membrane h+-ATPases to regulate stomatal apertures during pathogen attack. PLos Biol. 7, e1000139. doi: 10.1371/journal.pbio.1000139 19564897 PMC2694982

[B41] Ludewig F. Flugge U. I. (2013). Role of metabolite transporters in source-sink carbon allocation. Front. Plant Sci. 4, 231. doi: 10.3389/fpls.2013.00231 23847636 PMC3698459

[B42] Lushchak V. I. (2011). Adaptive response to oxidative stress: Bacteria, fungi, plants and animals. Comp. Biochem. Phys. C 153, 175–190. doi: 10.1016/j.cbpc.2010.10.004 20959147

[B43] Lu D. P. Wu S. J. Gao X. Q. Zhang Y. L. Shan L. B. He P. (2010). A receptor-like cytoplasmic kinase, BIK1, associates with a flagellin receptor complex to initiate plant innate immunity. P Natl. Acad. Sci. U.S.A. 107, 496–501. doi: 10.1073/pnas.0909705107 PMC2806711 20018686

[B44] Mackey D. Belkhadir Y. Alonso J. M. Ecker J. R. Dangl J. L. (2003). Arabidopsis RIN4 is a target of the type III virulence effector AvrRpt2 and modulates RPS2-mediated resistance. Cell 112, 379–389. doi: 10.1016/S0092-8674(03)00040-0 12581527

[B45] Mackey D. Holt B. F. Wiig A. Dangl J. L. (2002). RIN4 interacts with pseudomonas syringae type III effector molecules and is required for RPM1-mediated resistance in arabidopsis. Cell 108, 743–754. doi: 10.1016/S0092-8674(02)00661-X 11955429

[B46] Melotto M. Underwood W. Koczan J. Nomura K. He S. Y. (2006). Plant stomata function in innate immunity against bacterial invasion. Cell 126, 969–980. doi: 10.1016/j.cell.2006.06.054 16959575

[B47] Mersmann S. Bourdais G. Rietz S. Robatzek S. (2010). Ethylene signaling regulates accumulation of the FLS2 receptor and is required for the oxidative burst contributing to plant immunity. Plant Physiol. 154, 391–400. doi: 10.1104/pp.110.154567 20592040 PMC2938167

[B48] Miya A. Albert P. Shinya T. Desaki Y. Ichimura K. Shirasu K.. (2007). CERK1, a LysM receptor kinase, is essential for chitin elicitor signaling in arabidopsis. P Natl. Acad. Sci. U.S.A. 104, 19613–19618. doi: 10.1073/pnas.0705147104 PMC2148337 18042724

[B49] Modareszadeh M. Bahmani R. Kim D. G. Hwang S. (2020). CAX3 (cation/proton exchanger) mediates a cd tolerance by decreasing ROS through Ca elevation in arabidopsis. Plant Mol. Biol, 105, 115–132. doi: 10.1007/s11103-020-01072-1 32926249

[B50] Nasir K. Takahashi Y. Ito A. Saitoh H. Matsumura H. Kanzaki H.. (2005). High-throughput in planta expression screening identifies a class II ethylene-responsive element binding factor-like protein that regulates plant cell death and non-host resistance. Plant J. 43, 491–505. doi: 10.1111/j.1365-313X.2005.02472.x 16098104

[B51] Oliva R. Quibod I. L. (2017). Immunity and starvation: New opportunities to elevate disease resistance in crops. Curr. Opin. Plant Biol. 38, 84–91. doi: 10.1016/j.pbi.2017.04.020 28505583

[B52] Palm-Forster M. Eschen-Lippold L. Uhrig J. Scheel D. Lee J. (2017). A novel family of proline/serine-rich proteins, which are phospho-targets of stress-related mitogen-activated protein kinases, differentially regulates growth and pathogen defense in arabidopsis thaliana. Plant Mol. Biol. 95, 123–140. doi: 10.1007/s11103-017-0641-5 28755319 PMC5594048

[B53] Paparella C. Savatin D. V. Marti L. De Lorenzo G. Ferrari S. (2014). The arabidopsis LYSIN MOTIF-CONTAINING RECEPTOR-LIKE KINASE3 regulates the cross talk between immunity and abscisic acid responses. Plant Physiol. 165, 262–276. doi: 10.1104/pp.113.233759 24639336 PMC4012585

[B54] Qiu J. (2016). A study of mutual antagonism between EDS1 and transcription factor MYC2 in arabidopsis immunity ([PhD thesis]. (Cologne, Germany: Max Planck Institute for Plant Breeding Research).

[B55] Quiros C. F. Farnham M. W. (2011). "The genetics of brassica oleracea,". Eds. Schmidt R. Bancroft I. (New York, NY: Springer New York), 261–289.

[B56] Ranjan A. Westrick N. M. Jain S. Piotrowski J. S. Ranjan M. Kessens R.. (2019). Resistance against sclerotinia sclerotiorum in soybean involves a reprogramming of the phenylpropanoid pathway and up-regulation of antifungal activity targeting ergosterol biosynthesis. Plant Biotechnol. J. 17, 1567–1581. doi: 10.1111/pbi.13082 30672092 PMC6662107

[B57] Ritter C. Dangl J. L. (1996). Interference between two specific pathogen recognition events mediated by distinct plant disease resistance genes. Plant Cell 8, 251–257. doi: 10.1105/tpc.8.2.251 12239384 PMC161095

[B58] Rong W. Feng F. Zhou J. M. He C. Z. (2010). Effector-triggered innate immunity contributes arabidopsis resistance to xanthomonas campestris. Mol. Plant Pathol. 11, 783–793. doi: 10.1111/j.1364-3703.2010.00642.x 21029323 PMC6640269

[B59] Santos C. Nogueira F. Domont G. B. Fontes W. Prado G. S. Habibi P.. (2019). Proteomic analysis and functional validation of a brassica oleracea endochitinase involved in resistance to xanthomonas campestris. Front. Plant Sci. 10. doi: 10.3389/fpls.2019.00414 PMC6473119 31031780

[B60] Schroeder J. I. Neher R. E. (1987). Voltage dependence of k+channels in guard-cell protoplasts. P Natl. Acad. Sci. U.S.A. 84, 4108–4112. doi: 10.1073/pnas.84.12.4108 PMC305032 16593851

[B61] Seo P. J. Park C. M. (2010). MYB96-mediated abscisic acid signals induce pathogen resistance response by promoting salicylic acid biosynthesis in arabidopsis. New Phytol. 186, 471–483. doi: 10.1111/j.1469-8137.2010.03183.x 20149112

[B62] Seto D. Koulena N. Lo T. Menna A. Guttman D. S. Desveaux D. (2017). Expanded type III effector recognition by the ZAR1 NLR protein using ZED1-related kinases. Nat. Plants 3, 17027. doi: 10.1038/nplants.2017.27 28288096

[B63] Shimizu T. Nakano T. Takamizawa D. Desaki Y. Ishii-Minami N. Nishizawa Y.. (2010). Two LysM receptor molecules, CEBiP and OsCERK1, cooperatively regulate chitin elicitor signaling in rice. Plant J. 64, 204–214. doi: 10.1111/j.1365-313X.2010.04324.x 21070404 PMC2996852

[B64] Song K. Chen B. Cui Y. Zhou L. Chan K. G. Zhang H. Y.. (2022). The plant defense signal salicylic acid activates the RpfB-dependent quorum sensing signal turnover *via* altering the culture and cytoplasmic pH in the phytopathogen xanthomonas campestris. Mbio 13, e3621–e3644. doi: 10.1128/mbio.03644-21 PMC9040794 35254135

[B65] Song J. T. Koo Y. J. Seo H. S. Min C. K. Yang D. C. Kim J. H. (2008). Overexpression of AtSGT1, an arabidopsis salicylic acid glucosyltransferase, leads to increased susceptibility to pseudomonas syringae. Phytochemistry 69, 1128–1134. doi: 10.1016/j.phytochem.2007.12.010 18226820

[B66] Staswick P. E. Tiryaki I. (2004). The oxylipin signal jasmonic acid is activated by an enzyme that conjugates it to isoleucine in arabidopsis. Plant Cell 16, 2117–2127. doi: 10.1105/tpc.104.023549 15258265 PMC519202

[B67] Sun Q. Zhang E. Liu Y. Xu Z. Hui M. Zhang X.. (2021). Transcriptome analysis of two lines of brassica oleracea in response to early infection with xanthomonas campestris pv. campestris. Can. J. Plant Pathol. 43, 127–139. doi: 10.1080/07060661.2020.1775705

[B68] Tauzin A. S. Giardina T. (2014). Sucrose and invertases, a part of the plant defense response to the biotic stresses. Front. Plant Sci. 5, 293. doi: 10.3389/fpls.2014.00293 25002866 PMC4066202

[B69] van der Burgh A. M. Postma J. Robatzek S. Joosten M. (2019). Kinase activity of SOBIR1 and BAK1 is required for immune signalling. Mol. Plant Pathol. 20, 410–422. doi: 10.1111/mpp.12767 30407725 PMC6637861

[B70] Vega-alvarez C. Francisco M. Soengas P. (2021). Black rot disease decreases young brassica oleracea plants' biomass but has no effect in adult plants. Agronomy-Basel 11 (3), 569. doi: 10.3390/agronomy11030569

[B71] Wang G. X. Roux B. Feng F. Guy E. Li L. Li N. N.. (2015). The decoy substrate of a pathogen effector and a pseudokinase specify pathogen-induced modified-self recognition and immunity in plants. Cell Host Microbe 18, 285–295. doi: 10.1016/j.chom.2015.08.004 26355215

[B72] White F. F. Potnis N. Jones J. B. Koebnik R. (2009). The type III effectors of xanthomonas. Mol. Plant Pathol. 10, 749–766. doi: 10.1111/j.1364-3703.2009.00590.x 19849782 PMC6640274

[B73] Williams P. H. (1980). Black rot: A continuing threat to world crucifers. Plant Dis. 64, 736–742.

[B74] Willmann R. Lajunen H. M. Erbs G. Newman M. A. Kolb D. Tsuda K.. (2011). Arabidopsis lysin-motif proteins LYM1 LYM3 CERK1 mediate bacterial peptidoglycan sensing and immunity to bacterial infection. P Natl. Acad. Sci. U.S.A. 108, 19824–19829. doi: 10.1073/pnas.1112862108 PMC3241766 22106285

[B75] Wu J. Deng Y. Hu J. H. Jin C. Z. Zhu X. W. Li D. F. (2020). Genome-wide analyses of direct target genes of an ERF11 transcription factor involved in plant defense against bacterial pathogens. Biochem. Bioph. Res. Co 532, 76–81. doi: 10.1016/j.bbrc.2020.07.073 32828541

[B76] Xiang L. Le Roy K. Bolouri-Moghaddam M. R. Vanhaecke M. Lammens W. Rolland F.. (2011). Exploring the neutral invertase-oxidative stress defence connection in arabidopsis thaliana. J. Exp. Bot. 62, 3849–3862. doi: 10.1093/jxb/err069 21441406 PMC3134342

[B77] Yamada K. Saijo Y. Nakagami H. Takano Y. (2016). Regulation of sugar transporter activity for antibacterial defense in arabidopsis. Science 354, 1427–1430. doi: 10.1126/science.aah5692 27884939

[B78] Yang C. Li W. Cao J. D. Meng F. W. Yu Y. Q. Huang J. K.. (2017). Activation of ethylene signaling pathways enhances disease resistance by regulating ROS and phytoalexin production in rice. Plant J. 89, 338–353. doi: 10.1111/tpj.13388 27701783

[B79] Yang C. C. Wu P. F. Yao X. H. Sheng Y. Zhang C. C. Lin P.. (2022). Integrated transcriptome and metabolome analysis reveals key metabolites involved in camellia oleifera defense against anthracnose. Int. J. Mol. Sci. 23 (1) : 536. doi: 10.3390/ijms23010536 35008957 PMC8745097

[B80] Yin K. Q. Qiu J. L. (2019). Genome editing for plant disease resistance: Applications and perspectives. Philos. T. R. Soc. B. 374 (1767) , 20180322. doi: 10.1098/rstb.2018.0322 PMC6367152 30967029

[B81] Yuan M. H. Jiang Z. Y. Bi G. Z. Nomura K. Liu M. H. Wang Y. P.. (2021). Pattern-recognition receptors are required for NLR-mediated plant immunity. Nature 592, 105. doi: 10.1038/s41586-021-03316-6 33692546 PMC8016741

[B82] Zhang W. Jiang L. H. Huang J. Ding Y. Q. Liu Z. B. (2020). Loss of proton/calcium exchange 1 results in the activation of plant defense and accelerated senescence in arabidopsis. Plant Sci. 296, 110472. doi: 10.1016/j.plantsci.2020.110472 32540002

[B83] Zhang Z. B. Liu Y. N. Huang H. Gao M. H. Wu D. Kong Q.. (2017). The NLR protein SUMM2 senses the disruption of an immune signaling MAP kinase cascade *via* CRCK3. EMBO Rep. 18, 292–302. doi: 10.15252/embr.201642704 27986791 PMC5286374

[B84] Zhang J. Li W. Xiang T. T. Liu Z. X. Laluk K. Ding X. J.. (2010). Receptor-like cytoplasmic kinases integrate signaling from multiple plant immune receptors and are targeted by a pseudomonas syringae effector. Cell Host Microbe 7, 290–301. doi: 10.1016/j.chom.2010.03.007 20413097

[B85] Zhang H. B. Li A. Zhang Z. J. Huang Z. J. Lu P. L. Zhang D. Y.. (2016). Ethylene response factor TERF1, regulated by ETHYLENE-INSENSITIVE3-like factors, functions in reactive oxygen species (ROS) scavenging in tobacco (Nicotiana tabacum l.). Sci. Rep.-Uk 6, 29948. doi: 10.1038/srep29948 PMC4951782 27435661

[B86] Zhang C. Lv M. Yin W. Dong T. Chang C. Miao Y.. (2018). Xanthomonas campestris promotes diffusible signal factor (DSF) biosynthesis and pathogenicity by utilizing glucose and sucrose from host plants. Mol. Plant Microbe Interact. 32, 157–166. doi: 10.1094/MPMI-07-18-0187-R 30156480

[B87] Zhang Y. X. Xu S. H. Ding P. T. Wang D. M. Cheng Y. T. He J.. (2010). Control of salicylic acid synthesis and systemic acquired resistance by two members of a plant-specific family of transcription factors. P Natl. Acad. Sci. U.S.A. 107, 18220–18225. doi: 10.1073/pnas.1005225107 PMC2964219 20921422

[B88] Zhao H. B. Xu L. F. Su T. Jiang Y. Hu L. Y. Ma F. W. (2015). Melatonin regulates carbohydrate metabolism and defenses against pseudomonas syringae pv. tomato DC3000 infection in arabidopsis thaliana. J. Pineal. Res. 59, 109–119. doi: 10.1111/jpi.12245 25958775

[B89] Zheng X. Y. Spivey N. W. Zeng W. Q. Liu P. P. Fu Z. Q. Klessig D. F.. (2012). Coronatine promotes pseudomonas syringae virulence in plants by activating a signaling cascade that inhibits salicylic acid accumulation. Cell Host Microbe 11, 587–596. doi: 10.1016/j.chom.2012.04.014 22704619 PMC3404825

[B90] Zheng X. Xing J. H. Zhang K. Pang X. Zhao Y. T. Wang G. Y.. (2019). Ethylene response factor ERF11 activates BT4 transcription to regulate immunity to pseudomonas syringae. Plant Physiol. 180, 1132–1151. doi: 10.1104/pp.18.01209 30926656 PMC6548261

