# Supplementary figures and images for: Integrated metabolome and transcriptome analysis reveals salicylic acid and flavonoid pathways’ key roles in cabbage’s defense responses to Xanthomonas campestris pv. campestris
Source: Front Plant Sci. 2022 Oct 31;13:1005764. doi: 10.3389/fpls.2022.1005764 (PMC9659849; doi:10.3389/fpls.2022.1005764)

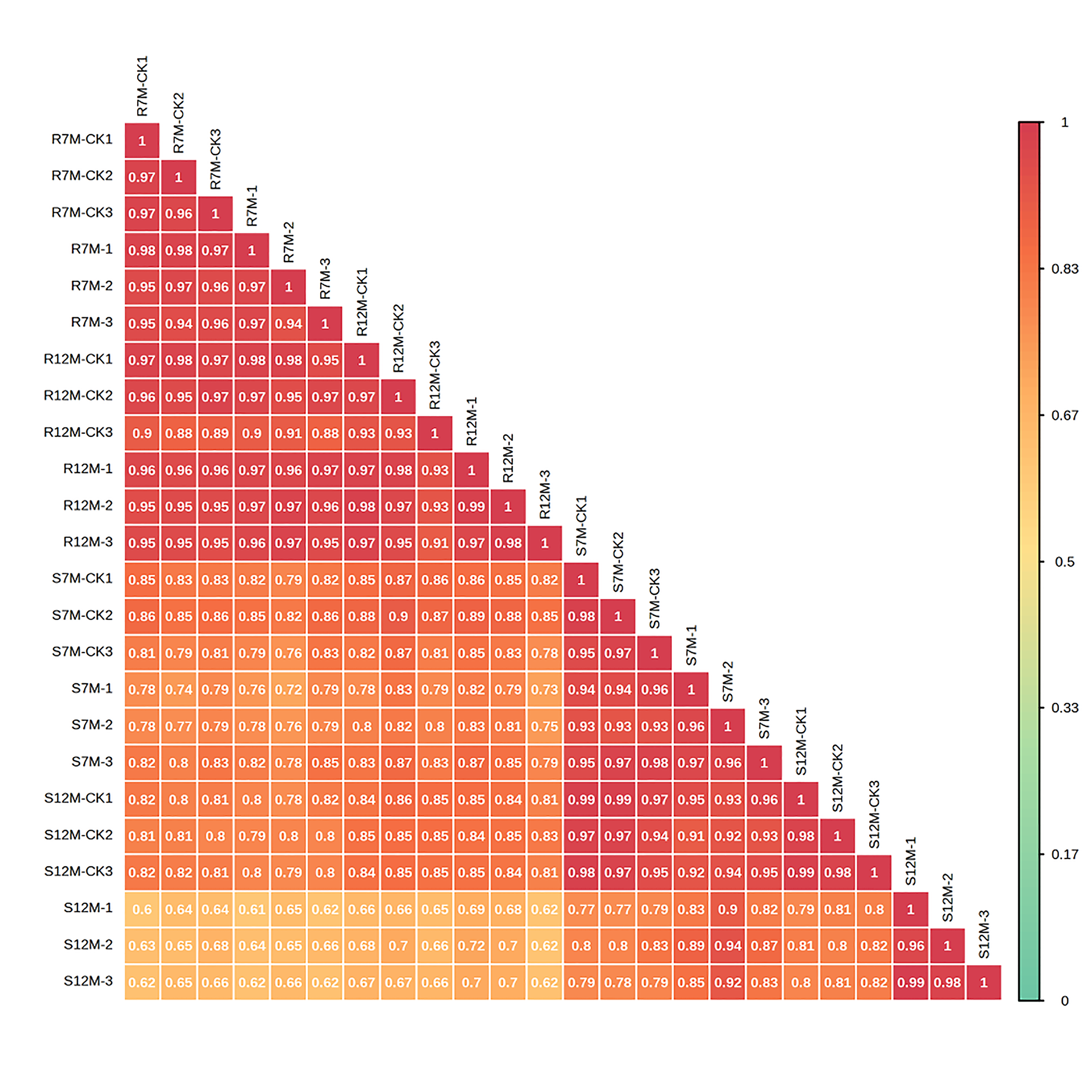

Supplement: Supplementary Figure 1 — Correlation analysis of the variance-stabilized estimated raw counts of differentially accumulated metabolites. [file Image_1.tif]

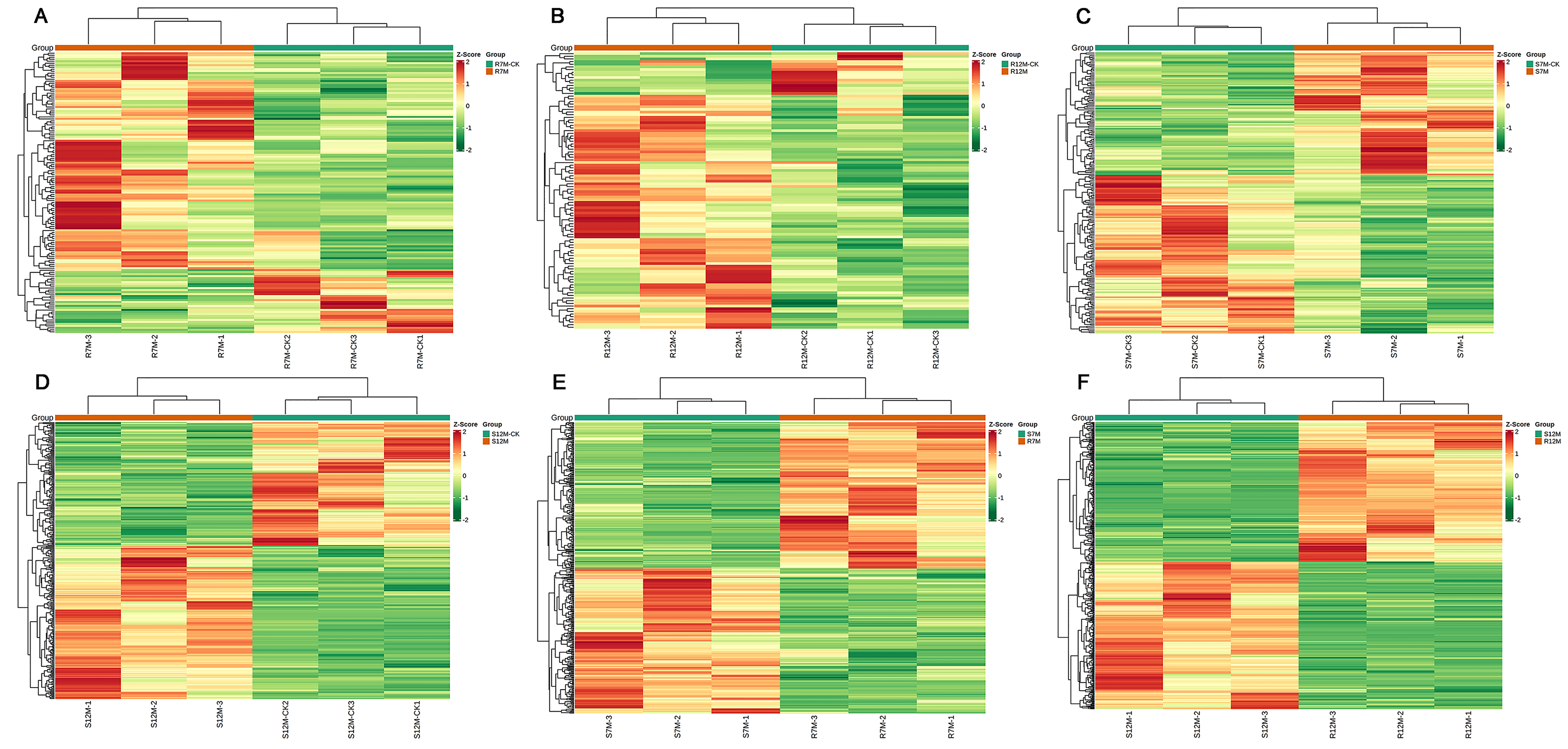

Supplement: Supplementary Figure 2 — Heatmap visualization of identified metabolites in comparisons. (A): R7M-CK vs R7M; (B): R12M-CK vs R12M; (C): S7M-CK vs S7M; (D): S12M-CK vs S12M; (E): S7M vs R7M; (F): S12M vs R12M. [file Image_2.tif]

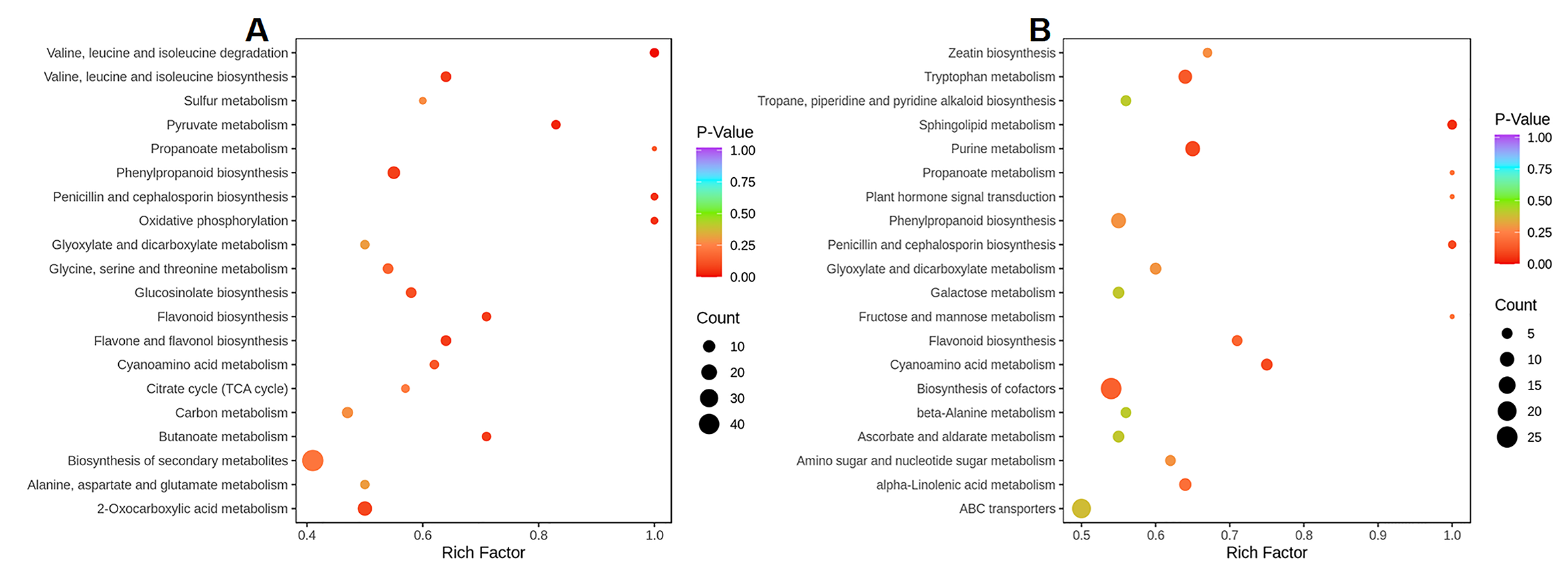

Supplement: Supplementary Figure 3 — Top 20 KEGG enrichment pathway of identified DAMs in comparisons. (A): S7M vs R7M; (B): S12M vs R12M. [file Image_3.tif]

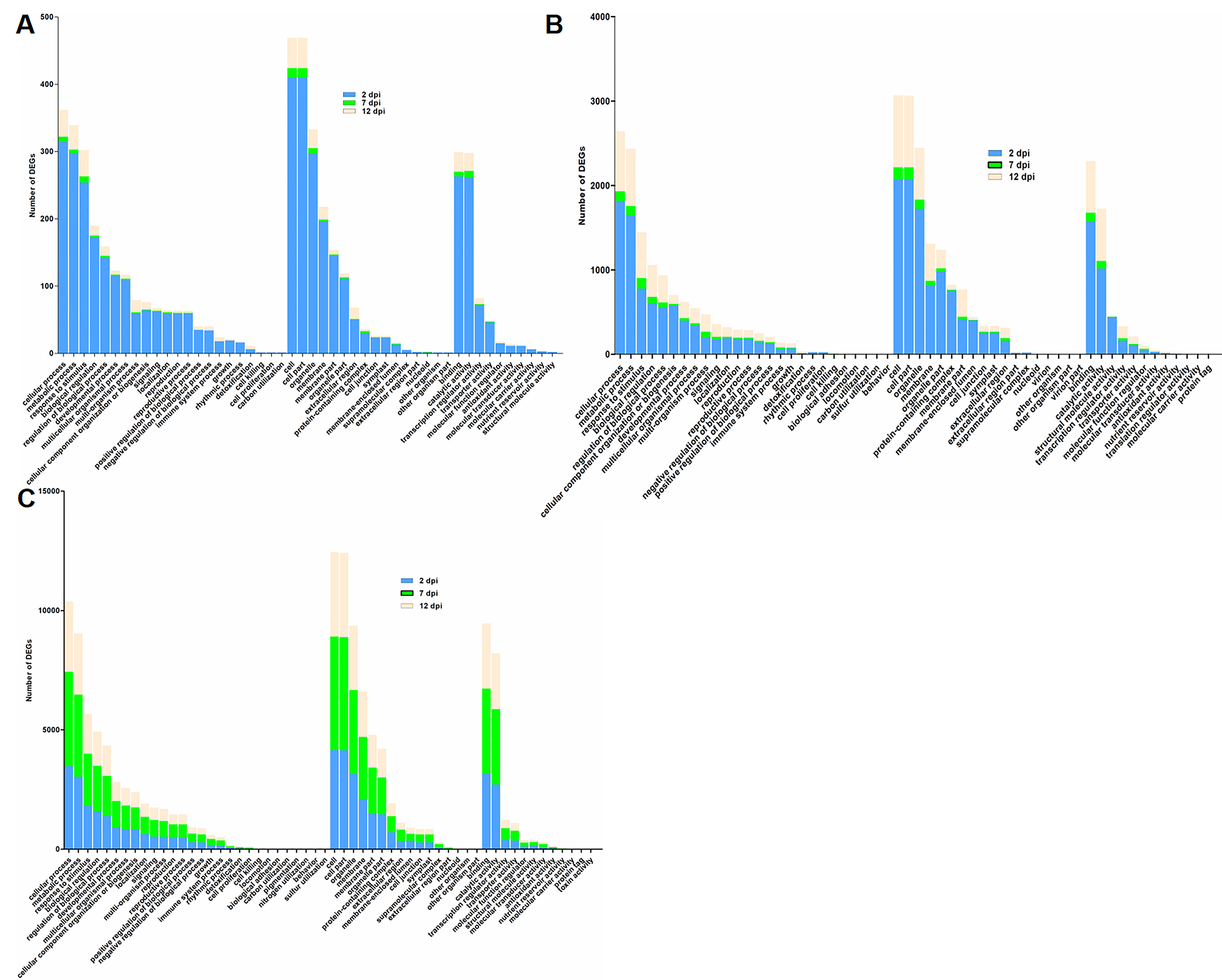

Supplement: Supplementary Figure 4 — GO assignment of identified DEGs in comparisons. (A): Comparison between control and inoculated resistant line. (B): Comparison between control and inoculated susceptible line. (C): Comparison between inoculated resistant and susceptible line. [file Image_4.tif]

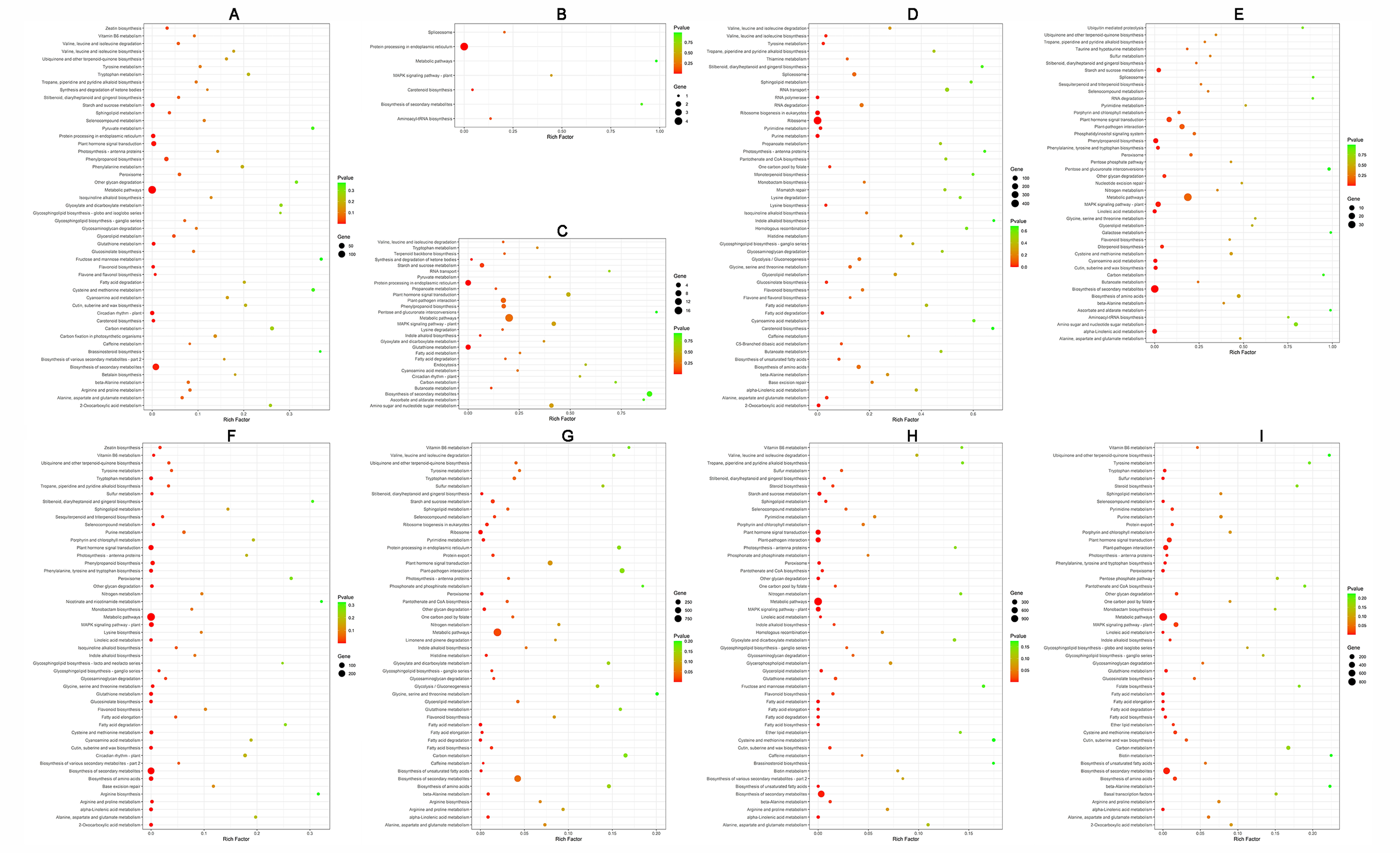

Supplement: Supplementary Figure 5 — Top 50 enriched KEGG pathways at all nine comparisons. (A): R2T-CK vs R2T; (B): R7T-CK vs R7T; (C): R12T-CK vs R12T; (D): S2T-CK vs S2T; (E): S7T-CK vs S7T; (F): S12T-CK vs S12T; (G): S2T vs R2T; (H): S7T vs R7T; (I): S12T vs R12T. [file Image_5.tif]

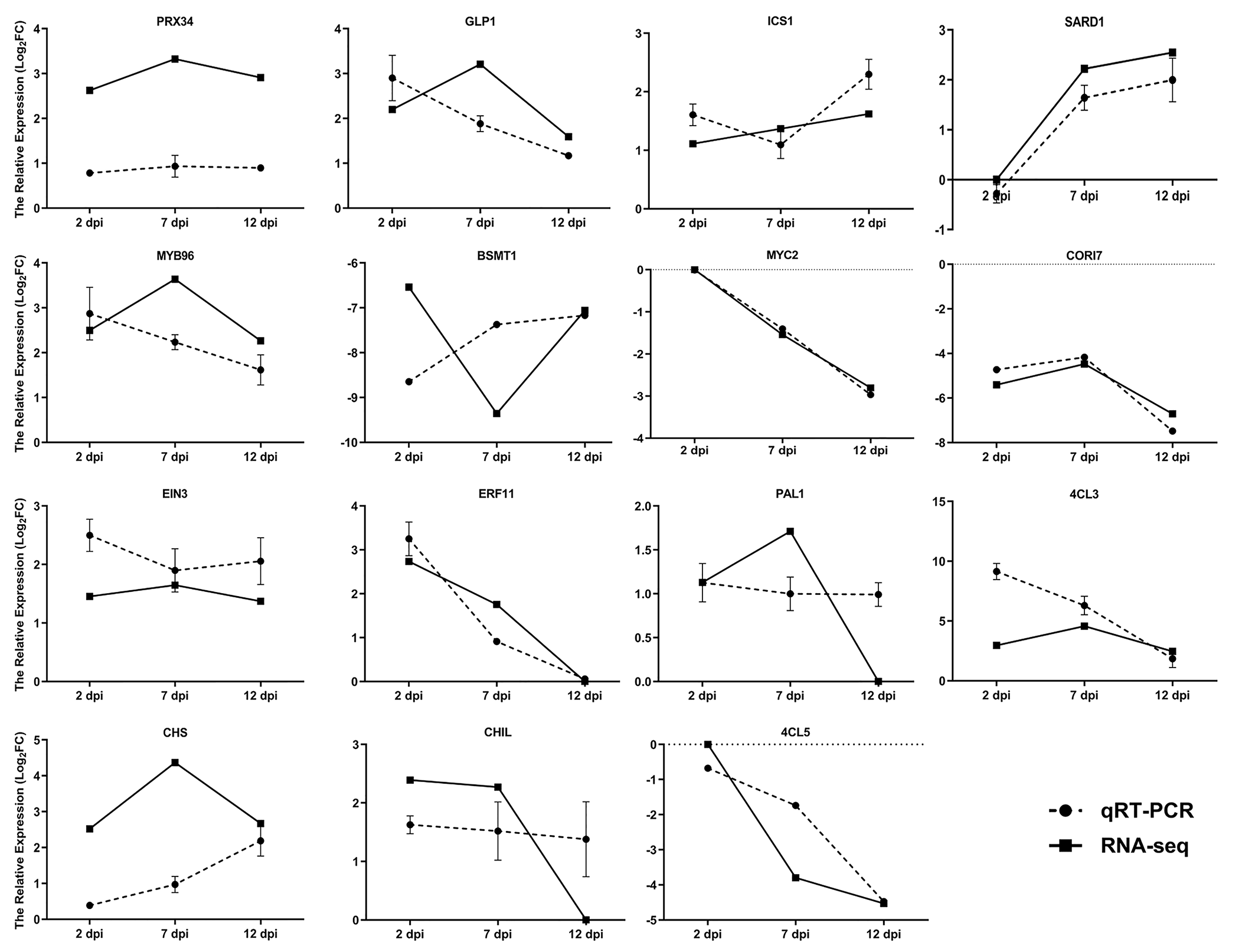

Supplement: Supplementary Figure 6 — Confirmation of expression profiles of select DEGs in R line compared with S line using qRT-PCR. [file Image_6.tif]
